# Prostate Cancer Research in Ghana: A Scoping Review of Research Direction and Priorities

**DOI:** 10.3390/cancers18203246

**Published:** 2026-10-09

**Authors:** Isaac Amoah, Hannah Ayettey Anie, Daniel Tetteh, Vanessa Adu Sarpong, Kwaku Addai Arhin Appiah, Phyllis Tawiah

**Affiliations:** 1 The Ghana integRative Approach to Cancer rEsearch (GRACE) Program, Korle Bu Teaching Hospital, Accra P.O. Box KB 369, Ghana; h.ayettey@kbth.gov.gh; 2 Department of Biochemistry and Biotechnology, Kwame Nkrumah University of Science and Technology, Kumasi 00233, Ghana; dtetteh26@st.knust.edu.gh (D.T.); vadusarpong@st.knust.edu.gh (V.A.S.); 3 National Radiotherapy Oncology and Nuclear Medicine Centre, Korle Bu Teaching Hospital, Accra P.O. Box KB 369, Ghana; 4 Department of Surgery, School of Medical Sciences, College of Health Sciences, Kwame Nkrumah University of Science and Technology, Kumasi 00233, Ghana; kwaku.appiah@knust.edu.gh; 5 Department of Medicine, School of Medical Sciences, College of Health Sciences, Kwame Nkrumah University of Science and Technology, Kumasi 00233, Ghana; phytawiah@knust.edu.gh

**Keywords:** digital rectal examination, Ghanaian men, prostate cancer, prostate neoplasm, prostate-specific antigen

## Abstract

**Background**: In Ghana, prostate cancer (PCa) was the second most frequently diagnosed cancer among men in 2022, after liver cancer. Despite increasing research interest, evidence needed to guide PCa research priorities remains fragmented. This scoping review aimed to highlight research priorities and direction on PCa in Ghana by identifying thematic areas, gaps and potential areas for future research. **Methods**: The scoping review was conducted in accordance with the Preferred Reporting Items for Systematic Reviews and Meta-Analyses Extension for Scoping Reviews checklist (PRISMA-ScR). A systematic search for relevant articles was conducted on four prominent databases: PubMed, Cochrane Library, Scopus, and ScienceDirect. **Results**: Data from eligible studies (*n* = 47) were synthesized across 15 thematic domains, including awareness, diagnosis and treatment approaches, genetics, dietary and metabolic factors, and pharmacological interventions. PCa awareness was high in some populations, but knowledge of specific disease features was variable, while screening uptake remained low. PSA was the most frequently assessed diagnostic marker, commonly evaluated alongside DRE and biopsy. Genomic studies identified potentially relevant genetic and molecular variants, although the evidence remains limited and requires further validation. Treatment patterns varied by disease stage and were also constrained by access- and resource-related factors. Epidemiological estimates differed by study design and population. **Conclusions**: PCa research in Ghana is broad but uneven, with significant gaps in early detection, population-level data, genomic validation and equitable access to care. Improving timely diagnosis and access to diagnostic and treatment services is important, while population-wide screening requires further evaluation of benefits, harms, cost-effectiveness, and health system capacity.

## Simple Summary

Prostate cancer is one of the most common cancers affecting men in Ghana, yet the available evidence is fragmented, with no comprehensive overview of the country’s research landscape. This scoping review maps the existing literature on prostate cancer among Ghanaian men, covering public awareness, screening and early detection, genetic and dietary factors, diagnosis, treatment, and patient care. The review aimed to identify key research themes, summarize the available evidence, and highlight important gaps requiring further investigation. The findings indicate that although awareness is high, in-depth understanding of prostate cancer remains limited, screening uptake is low, and most studies conducted are in urban settings, leaving rural populations underrepresented. By bringing together the available evidence, this review provides a clearer understanding of prostate cancer research in Ghana and identifies priority areas for future research, policy development, and interventions aimed at improving early detection, treatment and outcomes for Ghanaian men.

## 1. Introduction

Globally, prostate cancer (PCa) is the fourth most commonly diagnosed cancer, behind only lung, female breast and colorectal cancer. According to the 2022 Global Cancer Observatory (GLOBOCAN) report on the analysis of cancer-specific estimates for 185 countries, PCa accounted for a total of 1,466,680 new cases worldwide [1]. This made PCa the second most frequently diagnosed cancer overall and the most common cancer among men [1]. Geographically, disparities in PCa incidence and mortality exist. Incidence rates of PCa are almost threefold higher in highly developed countries than in lower-resource settings (35.5 vs. 12.6 per 100,000 population) [1]. Conversely, the high incidence rate observed for developed countries corresponds to lower PCa mortality compared to those reported for lower-resource settings (6.6 vs. 7.3 per 100,000 population) [1]. This pattern is attributable to factors that include underdiagnosis, delayed presentation, limited access to early detection, and inadequate availability of timely and effective treatment, which continue to remain prevailing challenges in lower-resource settings, including Ghana [2].

In Ghana, according to the GLOBOCAN report, PCa ranked among the top six cancers for 2022, alongside breast, liver, cervical, non-Hodgkin lymphoma and ovarian cancers [3]. More broadly, it has been established that over 24,000 new cancer cases are recorded annually in the country, with PCa ranking highest among cancers affecting older Ghanaian men, and the second most frequently diagnosed cancer among men, after liver cancer, accounting for 2395 new cases (21.0% of all new cancer cases among men) [4]. A comparative study of cancer burden among males aged 35 to 75 years in Ghana suggests that PCa accounts for a substantial proportion of cancer-related morbidity and mortality among Ghanaian men, particularly those aged 55 years and above [5]. In 2020, a systematic review on the incidence and mortality associated with prostate cancer in Ghana compared to other gynaecological cancers in the country was conducted. The author reported that the fatality rate for prostate cancer was high compared to breast and cervical cancer, with only 25% of advanced-diagnosed Ghanaian men surviving annually [6]. At the same time, awareness and uptake of screening remain low, while context-specific, evidence-based strategies for prevention and the management of advanced prostate cancer are still inadequately developed and implemented [7].

In response to the increasing burden of PCa, research conducted in Ghana has expanded across several domains, including assessing the burden, risk factors and biomarkers of the disease [8], screening and diagnosis, treatment outcomes [9], prognostic modelling [10], molecular and genetic epidemiology [11], and patient experiences [12]. These studies have generated important evidence relevant to the Ghanaian context. However, there is currently no comprehensive overview of the scope, characteristics and distribution of prostate cancer research in Ghana. The literature remains fragmented, with findings dispersed across diverse research themes, institutions and study designs. The absence of a systematic synthesis limits understanding of research trends, methodological patterns, areas of concentration and persistent evidence gaps, thereby constraining efforts to establish research priorities and guide evidence-informed policy, clinical practice, and future investigations.

The use of scoping reviews to consolidate PCa evidence in other Sub-Saharan African countries has been reported in other studies. For example, a recent scoping review and meta-analysis of prostate cancer detection and management in Nigeria synthesized evidence from 48 studies. The review highlighted that most of the selected studies were conducted within single states and were characterized by limited inter-institutional and multidisciplinary collaboration, highlighting important gaps in the geographical coverage and coordination of prostate cancer research [13]. Similar evidence-mapping and scoping exercises have been conducted, or proposed, to examine prostate cancer burden-reduction strategies across Sub-Saharan Africa, including region-specific assessments such as those undertaken in South Africa’s Eastern Cape Province [14]. Despite these advances, no dedicated synthesis has systematically mapped the landscape of prostate cancer research focused on Ghana. This represents an important gap in the literature, particularly given Ghana’s evolving population-based cancer surveillance system, its prominence as a key setting for ancestry-related prostate cancer research, and the substantial clinical and public health burden that prostate cancer places on Ghanaian men [5,15,16].

A scoping review was considered the most appropriate approach for addressing this evidence gap because the objective was to map the breadth and characteristics of prostate cancer research in Ghana rather than to answer a narrowly defined clinical question or estimate the effect of a specific intervention. Unlike systematic reviews that commonly focus on a specific question using predefined outcomes and, where appropriate, quantitative synthesis, scoping reviews are suited to examining heterogeneous bodies of literature and identifying how evidence is distributed across study designs, populations, settings, and research themes [17]. This was particularly relevant to prostate cancer research in Ghana, where the available studies span diverse areas, including awareness, screening, diagnosis, treatment, psychosocial outcomes, genomics, nutrition, and experimental pharmacological research. The approach therefore enabled us to map the major thematic areas investigated, describe the geographical and methodological distribution of the evidence, and identify topics and populations that remain insufficiently studied. Accordingly, this scoping review addresses the following question: What is known about the research priorities and direction of prostate cancer research among Ghanaian men? In answering this question, the scoping review aims to identify the principal thematic areas that have characterized PCa research in Ghana to date and propose future directions for investigation that remain insufficiently explored.

## 2. Materials and Methods

This scoping review was conducted in adherence to the Preferred Reporting Items for Systematic Reviews and Meta-Analyses Extension for Scoping Reviews checklist (PRISMA-ScR) [18] (Figure 1) and has not been registered. The completed PRISMA-ScR is provided as Supplementary Table S1. A scoping review was adopted in order to provide a comprehensive overview of the scope of work carried out and identify the research direction and priorities on PCa research in Ghana.

A systematic search for studies on PCa in Ghana was carried out on the databases PubMed, Cochrane Library, ScienceDirect, and Scopus from inception up to 20th November 2025. The search term used for PubMed and Cochrane Library was: (prostate cancer OR malignant prostate neoplasm* OR malignant prostate tumor OR malignant prostate tumour) AND (Ghana OR Ashanti region OR Kumasi OR Greater Accra region OR Accra OR Central region OR Cape Coast OR Eastern region OR Koforidua OR Volta region OR Ho OR Western region OR Sekondi-Takoradi OR Sekondi OR Takoradi OR Northern region OR Tamale OR Upper East region OR Bolgatanga OR Upper West region OR Wa OR Ahafo region OR Goaso OR Bono East region OR Techiman OR Oti region OR Dambai OR Savannah region OR Damongo OR North East region OR Nalerigu OR Western North region OR Sefwi Wiawso OR Bono region OR Sunyani). ScienceDirect and Scopus employed the search term: (prostate cancer OR malignant prostate neoplasm OR malignant prostate tumor OR malignant prostate tumour) AND (Ghana).

Three independent authors, I.A., D.T. and V.A.S., participated in the study selection and verification process. Following duplicate removal, title and abstract screening was independently undertaken against the eligibility criteria, after which potentially eligible articles underwent independent full-text assessment by the authors. In instances where there was a disagreement regarding eligibility at either stage between the authors, a consensus was established through discussion with author I.A. No search filters were applied. The obtained results from the article search were exported to ENDNOTE^®^ X7.8 (Bld 11583) library. The articles were screened for eligibility, and those that met the inclusion criteria were selected for this scoping review. No language filter was applied to the search itself, but non-English studies were excluded during eligibility screening. No lower publication date limit was applied; studies published from database inception to 20 November 2025 were all considered.

The inclusion criteria for this scoping review included: (i) studies conducted in Ghana, or studies in which the study population consisted of Ghanaian participants, or studies that utilized genomic or clinical dataset from Ghanaian individuals, (ii) studies that focused specifically on PCa, encompassing any aspect of the disease, including knowledge, perceptions, awareness, diagnosis, screening, treatment, nutrition, prognosis, genomics and/or clinical epidemiology, (iii) peer-reviewed original research articles, (iv) articles published in the English language, (v) studies available as full-text publications, accessible through institutional subscriptions and open access repositories, and (vi) no restrictions were placed on study design because the scoping review sought to capture the breadth of prostate cancer research in Ghana.

The exclusion criteria included: (i) book chapters, commentaries, letters to editors, conference abstracts or proceedings, narrative or systematic reviews, (ii) studies with a primary Sub-Saharan African or multi-country scope were excluded where Ghana-specific participants/data cannot be separately identified or extracted, (iii) studies addressing other urological conditions, other malignancies or disease conditions unrelated to prostate, (iv) studies whose primary objective was to compare prostate cancer outcomes, treatment responses, genomic profiles, or epidemiological patterns between Ghanaian or African populations and non-African populations, as the review sought to map evidence generated within the Ghanaian context rather than synthesize comparative evidence across populations, (v) studies conducted exclusively in non-Ghanaian populations, with no identifiable Ghanaian participant subset or data contribution.

The search was restricted to peer-reviewed original research articles indexed in the selected electronic databases. Grey literature sources, including dissertations and theses, government and institutional reports, conference abstracts or proceedings, and other unpublished materials, were not systematically searched. Cancer registry data were eligible where they were reported within peer-reviewed original research articles that met the inclusion criteria; standalone unpublished cancer registry reports were not separately searched.

The authors developed a form for extracting data on the outcomes of interest from the selected articles. The parameters extracted from the selected articles include the name of the first author and year of publication, characteristics of the study participants, study design, sample size, stage of the PCa, and the objective of the study and the study findings. In instances where an intervention was carried out, the duration of the intervention, dosage/amount of the intervention, and form of the intervention were additionally indicated. Following the data extraction, the included studies were reviewed and synthesized thematically. The thematic categories were not predefined; instead, they emerged inductively from the included studies based on similarities in their primary objectives, outcomes assessed, and main research focus. Studies addressing related aspects of prostate cancer research were grouped into common thematic domains. This process resulted in the fifteen thematic domains used to organize and present the findings of the review.

Research priorities were identified from the thematic synthesis by examining the distribution of evidence and the knowledge gaps within each of the 15 domains. Themes with limited evidence, underrepresented populations or geographic settings, methodological limitations, or unresolved research questions were considered areas requiring further investigation. These priorities were therefore derived from the mapped evidence rather than through stakeholder consultation or a formal ranking exercise.

This scoping review addressed the question: What is known about the research priorities and direction of PCa in Ghanaian men? This question is to guide the authors towards identifying the thematic areas in PCa research in Ghana and to propose future directions that can be explored for PCa research amongst Ghanaian men.

## 3. Results

### 3.1. Study Selection

A total of 10,987 articles were obtained from the searches conducted on PubMed (*n* = 8807), Cochrane Library (*n* = 186), ScienceDirect (*n* = 1100), and Scopus (*n* = 894). Following the removal of 228 duplicate records, a total of 10,759 articles were obtained for title and abstract screening. Of these, 10,587 records were excluded, resulting in 172 papers that were assessed for eligibility by applying the inclusion and exclusion criteria to their full text. A total of 47 study reports were identified as eligible and subsequently retained for this scoping review (Figure 1). The findings of this scoping review were grouped according to 15 thematic areas and presented in Table 1, Table 2, Table 3, Table 4, Table 5, Table 6, Table 7, Table 8, Table 9, Table 10, Table 11, Table 12, Table 13, Table 14 and Table 15. The characteristics of all 47 included studies are summarized in Supplementary Table S2.

### 3.2. Geographic Distribution of Prostate Cancer Research in Ghana

The geographic distribution of PCa research in Ghana was markedly uneven. Among the 47 unique studies represented in the evidence tables after application of the revised eligibility criteria, 25 of the studies were conducted in or included participants from the Greater Accra Region, seven in the Ashanti Region, four in the Central Region, and three each in the Bono and Volta Regions. Five studies (*n* = 5) did not provide a sufficiently specific Ghanaian region in the extracted information. Study settings also showed a strong concentration in urban, municipal, or town-based locations. Thirty-seven studies (*n* = 37) reported identifiable cities, municipalities, or towns, including Accra, Kumasi, Cape Coast, Sunyani, Ejisu, Agogo, Wenchi, Ayigya, Ho, Hohoe, and Sogakope. No included study explicitly identified its study setting as rural. The remaining 10 studies reported region-wide or insufficiently specified geographic information and were thus not classified.

Greater Accra was the principal research hub, with much of the clinical evidence originating from tertiary and specialist facilities in Accra, particularly Korle Bu Teaching Hospital and associated institutions. Ashanti Region represented the second major research centre, with studies conducted mainly in Kumasi and surrounding municipalities. Comparatively fewer studies were conducted in the Central, Bono, and Volta Regions. No eligible published studies were identified from the other regions. The absence of explicitly rural studies and the lack of evidence from 11 of Ghana’s 16 administrative regions demonstrate substantial geographic gaps in the current evidence base.

Overall, the geographical distribution demonstrates a marked urban concentration of prostate cancer research, with much of the available evidence generated from tertiary referral centres, Korle Bu Teaching Hospital in Accra and the Komfo Anokye Teaching Hospital in Kumasi, both located in southern Ghana. Consequently, important knowledge gaps remain regarding the epidemiology, screening practices, clinical management, and survivorship experiences of men residing in northern and rural regions of the country. Heavy dependence on studies conducted in the Greater Accra and Ashanti Regions limits the generalizability of findings to rural populations where healthcare infrastructure, health-seeking behaviour, and access to specialized oncology services differ considerably. Future research should deliberately incorporate rural populations and currently underrepresented regions to determine whether PCa awareness, screening practices, disease presentation, access to diagnosis and treatment, and patient outcomes differ from those documented in the better-studied urban centres.

### 3.3. Study Characteristics

The methodological approaches employed across the included studies were diverse, reflecting the broad scope of prostate cancer research conducted in Ghana. Cross-sectional studies constituted the largest proportion of the evidence base and were predominantly used to investigate awareness, screening behaviours, psychosocial outcomes, and healthcare utilization. Retrospective cohort studies frequently examined treatment outcomes, disease progression, and survival using hospital records, while prospective cohort studies evaluated clinical interventions and treatment effectiveness over time. Several qualitative studies explored the lived experiences of patients and caregivers, highlighting psychosocial challenges associated with diagnosis, treatment, and caregiving. A smaller number of methodological validation studies focused on developing culturally appropriate prostate cancer knowledge assessment tools, whereas laboratory-based and molecular studies investigated biomarkers, genetics, and disease mechanisms.

Sample sizes varied considerably according to study design and purpose, ranging from small qualitative studies involving approximately 10–12 participants to large clinical datasets involving several thousand participants. This heterogeneity reflects the different research questions addressed across the included literature and limits direct comparison among studies. Clinical and treatment-related studies were frequently conducted in tertiary hospitals and specialist oncology centres, whereas awareness and screening studies more commonly recruited participants from community, occupational, or population-based settings.

Overall, observational study designs predominated, with relatively few interventional studies or population-based longitudinal investigations. The predominance of observational and hospital-based research indicates that Ghanaian prostate cancer research remains largely descriptive. While these studies have substantially improved understanding of awareness, diagnosis and treatment patterns, relatively few intervention studies have evaluated strategies capable of improving screening uptake, early diagnosis, or patient outcomes. Small samples in some qualitative and experimental studies may also limit the transferability or generalizability of their findings. These methodological characteristics should therefore be considered when interpreting the evidence synthesized in this review.

## 4. Discussion

This review synthesizes evidence from 15 tables to provide a comprehensive appraisal of prostate cancer (PCa) in Ghana, spanning knowledge and awareness, screening practices, epidemiology, treatment outcomes, psychosocial burden, nutritional and genomic factors, pharmacological management, and health system constraints. Taken together, the findings depict a high-burden disease landscape characterized by late presentation, low screening uptake and systemic limitations, despite the presence of effective treatment modalities within tertiary care settings. Across the 15 tables, a clear pattern emerges in how prostate cancer research in Ghana is distributed, the types of participants included, and the contexts in which studies are conducted. These structural features are not merely descriptive; they fundamentally shape how the burden, presentation and management of prostate cancer are understood within the Ghanaian context. Interpretation of the findings should take account of the study populations and settings from which the evidence was generated. Many of the included studies were hospital-based, single-centre, or observational and therefore describe selected patient or community groups rather than the Ghanaian male population as a whole. Accordingly, findings from these studies are interpreted as study-specific observations, while population-level conclusions are drawn only where they are supported by population-based screening or cancer registry evidence with clearly defined denominators.

In terms of regional distribution, the body of evidence is heavily concentrated in a few urban and relatively well-resourced regions, most notably Greater Accra and Ashanti. Studies conducted in Accra and Kumasi dominate across nearly all thematic areas, including awareness, screening, diagnosis, treatment and prevalence. This concentration reflects the presence of major tertiary healthcare facilities and research institutions, such as Korle Bu Teaching Hospital and Komfo Anokye Teaching Hospital, which serve as key centres for oncology services and academic activity. While a small number of studies extend to regions such as Volta, large parts of the country, particularly northern and more rural regions, remain underrepresented or absent. This uneven geographical distribution suggests that the current evidence base is not fully representative of the national population but is instead shaped by the availability of diagnostic infrastructure and research capacity. Consequently, prostate cancer burden in underserved regions is likely underestimated, as limited access to screening and diagnostic services reduces case detection.

The characteristics of the study participants further reflect this structural imbalance and vary considerably depending on study design and setting. Across the studies, the participants are predominantly adult and older men, typically within the age range associated with increased prostate cancer risk. Community-based studies, particularly those focusing on awareness and screening, often include apparently healthy individuals drawn from the general population. These studies consistently reveal low levels of screening uptake, gaps in knowledge depth and the presence of undiagnosed disease. In contrast, hospital-based studies largely include patients who have already presented for care, many of whom exhibit advanced-stage disease at diagnosis. These clinical populations are more likely to have elevated prostate-specific antigen levels, higher Gleason scores and more aggressive disease phenotypes. The distinction between community and clinical populations is therefore critical: while the former provides insight into underlying risk and preventive behaviour, the latter reflects the consequences of delayed detection. Across both groups, socioeconomic and educational factors appear to influence health-seeking behaviour, awareness and access to care, further contributing to disparities in outcomes.

The variation in study settings across the included literature also has important implications for interpreting findings. A substantial proportion of studies are hospital-based, particularly those examining diagnosis, treatment and prevalence. These studies, typically conducted in tertiary referral centres, capture patients who have successfully navigated the healthcare system and are therefore more likely to present with advanced or symptomatic disease. As a result, hospital-based data tend to report higher prevalence proportions and greater disease severity, but are inherently subject to referral and selection biases. In contrast, population-based studies, though fewer in number, offer more generalizable estimates of incidence and disease distribution. The Kumasi Cancer Registry, for example, provides valuable population-level data, yet its limited geographic coverage highlights the absence of a comprehensive national cancer surveillance system. Community-based and screening studies represent another important setting, particularly for understanding awareness, preventive practices, and the hidden burden of disease. Finally, the inclusion of experimental laboratory-based studies, particularly those investigating natural products with cytotoxic effects on prostate cancer cell lines, reflects an emerging but still preliminary area of research. While these studies contribute to the scientific understanding of potential therapeutic agents, they remain preclinical and have not yet translated into clinical practice.

Taken together, the interplay between regional distribution, participant characteristics, and study setting reveals that the current understanding of prostate cancer in Ghana is shaped as much by structural and methodological factors as by underlying disease patterns. The concentration of studies in urban centres, the predominance of hospital-based data, and the limited scope of population-based research collectively contribute to an evidence base that may overrepresent advanced disease while underestimating true population burden. At the same time, the relative scarcity of longitudinal and nationally representative studies limits the ability to track trends and evaluate interventions over time.

*THEME 1: Knowledge, Awareness and Perceptions of PCa*

The findings synthesized in Table 1 reveal a paradoxical pattern of relatively high awareness but inadequate knowledge and poor screening uptake of prostate cancer (PCa) among some populations of Ghanaian men. For clarity in interpreting the findings, awareness, disease knowledge, risk perception, and screening behaviour were treated as related but distinct concepts. Awareness refers to whether participants had heard of or recognized prostate cancer (PCa), whereas disease knowledge relates to understanding specific aspects of the disease, including its signs and symptoms, risk factors, screening methods, and treatment options. Risk perception refers to how individuals perceive their own susceptibility to PCa and the seriousness of the disease, while screening behaviour refers to whether they have actually undergone screening. The need to distinguish these dimensions is consistent with cancer awareness measurement frameworks, which assess knowledge of warning signs and risk factors separately from screening awareness, beliefs, and help-seeking behaviour [64]. Similarly, the Health Belief Model distinguishes perceived susceptibility, perceived severity, perceived benefits, and perceived barriers as separate factors that may influence preventive health behaviour [25].

Across the included studies, awareness levels were high in some of the populations surveyed. Osei Agyemang et al. [19] reported that 85.8% of the participants were aware of PCa, even though only 50.1% knew its signs and symptoms, 32% did not know its risk factors, and 54.1% could not identify a treatment method. This disconnect between awareness and action is consistent with evidence from other Sub-Saharan African settings, where awareness is often superficial and not accompanied by actionable health literacy [65,66]. Similarly, Asiamah-Asare and Ackumey [20] reported that 57.5% of male teachers had adequate overall knowledge of PCa, while only 37.5% knew about a screening test for the disease. These findings demonstrate that recognition of PCa as a disease should not be interpreted as equivalent to comprehensive knowledge of its presentation, risk factors, screening, and treatment. Similar distinctions have been reported across Sub-Saharan Africa, where inadequate knowledge, perceptions, attitudes, and beliefs have been identified as separate barriers to PCa screening [67]. Perceptions of PCa and screening also differed from general disease awareness. Yeboah-Asiamah et al. [21] found that 95% of participants considered PCa screening beneficial. This represents a favourable perception of screening rather than evidence of comprehensive disease awareness.

Importantly, misconceptions about PCa aetiology and transmission were prominent, reflecting deficiencies in health education. For example, 40.6% of participants attributed PCa to mobile phone radiation, while a proportion incorrectly associated it with sexual transmission [21]. These findings align with prior studies in low- and middle-income countries, where cultural beliefs and misinformation significantly distort disease understanding and influence health-seeking behaviour [68,69]. Another critical observation is the association between knowledge levels and perception. Participants with higher knowledge may demonstrate more positive perceptions toward PCa and screening [21]. Interestingly, Boafo et al. [25], using the Health Belief Model, found that men generally did not perceive themselves as susceptible to PCa, although 79.2% indicated that they would be willing to screen if screening was recommended by a healthcare provider. Thus, recognizing PCa or acknowledging the benefits of screening does not necessarily imply that individuals perceive themselves to be personally at risk. Comparatively, studies from high-income countries demonstrate a stronger alignment between awareness and screening behaviour, largely due to organized screening programmes, better health system access, and higher functional health literacy [68]. In contrast, the Ghanaian literature reports fragmented awareness dissemination, limited screening infrastructure, delayed diagnosis, and poor outcomes, although the available studies do not establish causal relationships among these factors [42,70]. Furthermore, the perception of PCa as a fatal disease (93.8%) [21] may have dual implications. While it may increase perceived severity, it can also induce fear-based avoidance, a phenomenon widely reported in the cancer epidemiology literature [71]. Fatalistic perceptions, inadequate disease knowledge, and late presentation were reported across the reviewed literature; however, the available observational evidence does not establish a causal relationship among these factors [19,21]. From a public health perspective, these findings underscore that awareness campaigns alone are insufficient. Instead, there is a need for comprehensive, culturally tailored health education interventions that move beyond awareness to improve functional and critical health literacy, correct misconceptions, and promote informed decision-making. Integration of prostate cancer education into primary healthcare systems and community-based outreach programmes may be particularly effective in bridging this gap.

In summary, the evidence suggests that while awareness of PCa in Ghana is relatively high, significant deficiencies in knowledge, persistent misconceptions, and low screening uptake remain major challenges. Addressing these may support informed help-seeking and appropriate early detection practices of PCa in the country.

*THEME 2: Tools for Assessing Knowledge, Awareness and Perceptions of PCa*

The evidence presented in Table 2 highlights a critical but often underemphasized dimension of prostate cancer (PCa) research in Ghana: the development, adaptation, and validation of culturally appropriate measurement tools for assessing knowledge, awareness, and perceptions. The studies by Wiafe et al. [9] and Wiafe et al. [22] collectively demonstrate both the limitations of existing assessment approaches and the methodological advancements required to improve data validity in this context. A key finding from the pilot study conducted by Wiafe et al. [9] is the poor performance of knowledge assessment items, with less than 25% and 20% of participants correctly responding to questions on signs/symptoms and risk factors, respectively. While this may initially be interpreted as low knowledge levels, it is equally indicative of instrument-related challenges, including issues of language, cultural framing, and contextual relevance. Standardized questionnaires developed in high-income settings often fail to capture nuanced understanding in African populations due to linguistic barriers and differences in health conceptualization [69,72].

Addressing this limitation, Wiafe et al. [22] undertook a psychometric validation of the Prostate Cancer Knowledge, Attitudes, and Beliefs Ghana (PCKAB-Gh) tool, a 41-item instrument developed using forward and backward translation into Akan (Asante Twi). The study reported a five-factor structure with evidence of construct validity, suggesting that the instrument can assess distinct domains of PCa-related knowledge, attitudes, and beliefs. This represents a significant methodological advancement, as psychometrically sound tools are essential for generating accurate epidemiological and behavioural data. The importance of culturally adapted tools is well supported in global health research. Measurement instruments that are not culturally validated often lead to systematic misclassification bias, thereby underestimating or overestimating true knowledge levels [68]. In Sub-Saharan Africa, where multilingualism and varying literacy levels are prevalent, the use of locally translated and validated instruments such as the PCKAB-Gh, enhances both internal validity and generalizability of findings [66].

Moreover, the inclusion of women in these studies [9,22] introduces an important dimension often overlooked in PCa research. Women frequently serve as health decision influencers within households, particularly in African settings. However, the findings indicate that women’s knowledge of PCa is substantially limited, suggesting that current awareness strategies may be insufficiently inclusive. This aligns with broader literature emphasizing the role of family and social networks in shaping men’s health-seeking behaviours, especially in patriarchal and community-oriented societies [65]. Another critical implication of these findings relates to measurement standardization across studies. The heterogeneity of tools used in earlier studies (such as structured questionnaires without validated scales) limits comparability and synthesis of evidence. The development of a validated instrument such as PCKAB-Gh provides an opportunity to standardize data collection across regions and populations in Ghana, thereby strengthening future systematic reviews and meta-analyses.

From a methodological standpoint, the validation process employed by Wiafe et al. [22], including factor analysis and reliability testing, aligns with best practices in psychometrics. However, further work is needed to assess test–retest reliability, responsiveness to interventions and cross-regional applicability within Ghana’s diverse ethnic landscape. Additionally, integration of such tools into national health surveys and screening programmes could significantly improve surveillance of PCa awareness and behavioural outcomes. In comparison to high-income countries, where validated instruments such as the Prostate Cancer Knowledge Scale (PCKS) and other standardized tools are routinely used, Ghana and similar settings are still in the early stages of tool development and validation [68]. This gap underscores the need for continued methodological investment in instrument design tailored to local contexts.

In conclusion, the findings from this theme underscore that accurate assessment of PCa awareness in Ghana is contingent upon the use of culturally appropriate, psychometrically validated tools. The development of the PCKAB-Gh tool represents a significant step forward; however, broader adoption and further validation are required to ensure reliable, comparable, and policy-relevant data. Strengthening measurement tools will ultimately enhance the effectiveness of awareness interventions and contribute to improved PCa outcomes in Ghana.

*THEME 3: Sources of PCa Awareness*

The findings in Table 3 provide important insights into the channels through which prostate cancer (PCa) information is disseminated in Ghana, revealing a communication landscape dominated by mass media and interpersonal networks, with relatively limited engagement from formal healthcare systems. Across the included studies, radio and television emerged as the primary sources of awareness, with 68% and 57% of participants citing television and radio, respectively [20], and similarly, 52.1% reporting radio as their main source [19]. This pattern reflects the broader media consumption trends in Ghana and Sub-Saharan Africa, where radio remains one of the most accessible and far-reaching communication tools, particularly in semi-urban and rural settings [66]. The prominence of these channels underscores their strategic importance for public health messaging, especially for population-level awareness campaigns.

However, despite the wide reach of mass media, the depth and accuracy of knowledge conveyed through these platforms appear limited, as evidenced in Theme 1, where high awareness did not translate into adequate understanding. This suggests that media-based health communication may prioritize exposure over comprehension, potentially contributing to the persistence of misconceptions about PCa [69]. Similar observations have been reported in other low- and middle-income countries, where mass media campaigns increase awareness but often fail to significantly improve health literacy or behavioural outcomes [68,73]. Interpersonal sources of information also play a notable role. In the study by Daniels et al. [23], health professionals (89%) were the most cited source, followed by friends (62%), television (61%), family members (39%), and the internet (48%). This contrasts with community-based studies where health professionals contributed minimally to awareness (as low as 4–4.6%) [19]. This discrepancy is likely attributable to differences in study populations, as Daniels et al. [23] focused on patients already engaged with the healthcare system, whereas other studies sampled the general population.

This divergence highlights a critical issue: that is, healthcare professionals are highly effective sources of information but are underutilized at the community level. In well-resourced settings, primary care providers play a central role in cancer education and screening advocacy. However, in Ghana, limited patient–provider interaction, workforce constraints, and a lack of structured screening programmes reduce opportunities for such engagement [72,74]. The relatively low contribution of digital and print media [19] suggests that health information dissemination is still largely traditional rather than digital-driven. This is notable given the increasing penetration of mobile technology in Ghana. Compared to high-income countries, where internet-based platforms and digital health campaigns are sources of cancer information, Ghana appears to lag in leveraging e-health interventions for PCa awareness [75].

Another important dimension is the role of social networks, including friends and family, which were reported as significant sources of information [23]. While these networks can facilitate information diffusion, they also pose a risk of propagating misinformation, particularly in contexts where baseline knowledge is low. This aligns with findings from Theme 1, where misconceptions about PCa aetiology were prevalent, potentially reflecting informal transmission of inaccurate health information. From a behavioural perspective, the dominance of passive information channels (such as radio and television), and over interactive and personalized sources such as health professionals, may contribute to the observed gap between awareness and screening uptake. The Health Belief Model (HBM) suggests that cues to action such as direct recommendations from healthcare providers are more likely to trigger behaviour change than general awareness messages [25]. Comparatively, in high-income settings, PCa awareness is often driven by organized screening campaigns, physician-led counselling and digital decision aids, which provide more tailored and actionable information [68,76]. The absence of such structured systems in Ghana contributes to fragmented and inconsistent information dissemination, limiting the effectiveness of awareness efforts.

Overall, the sources of PCa awareness in Ghana are predominantly mass media and informal social networks, with limited engagement from healthcare professionals and digital platforms. While radio and television offer extensive reach, their impact on deep knowledge acquisition and behaviour change appears constrained. Strengthening the role of healthcare providers, expanding digital health interventions, and improving the quality and consistency of media messaging are essential strategies for enhancing PCa awareness and promoting early detection practices in Ghana.

*THEME 4: Cost of PCa Treatment in Ghana*

The evidence summarized in Table 4 highlights the substantial economic burden associated with prostate cancer (PCa) diagnosis and management in Ghana, underscoring cost as a critical determinant of access to care, treatment choices, and ultimately, patient outcomes. The study by Wiafe et al. [24] provides one of the most comprehensive cost analyses in the Ghanaian context, demonstrating that the direct cost of diagnosing metastatic PCa (GHC 6876.00) is approximately four times higher than that of non-metastatic disease (GHC 1686.00). Given the high prevalence of late presentation in Ghana [19,42], these findings suggest that a substantial proportion of patients are subjected to this financial strain partly due to delayed detection.

Treatment costs further compound this burden. The estimated cost for radical prostatectomy and external beam radiotherapy (EBRT) was GHC 2150.00 each, while a 6-month course of androgen deprivation therapy (ADT) using goserelin cost approximately GHC 3000.00 [24]. When contextualized within Ghana’s average income levels and high out-of-pocket healthcare expenditure, these costs may represent a substantial financial burden for many households. This aligns with broader evidence from low- and middle-income countries, where financial toxicity is a major barrier to cancer care utilization and adherence [77,78,79]. A particularly concerning finding is the limited availability and variable pricing of PCa medications [24], which indicates significant inequities in drug access and affordability, likely driven by supply chain constraints, reliance on imports, and lack of price regulation mechanisms [80]. Similar challenges have been documented across Sub-Saharan Africa, where inconsistent drug availability leads to treatment interruptions and suboptimal clinical outcomes [81].

These economic barriers have important behavioural implications. As evidenced in Table 5, patients often opt for traditional medicine due to its perceived affordability, with 79.8% preferring it over orthodox care [26]. This suggests that cost is not merely a financial issue but also a determinant of healthcare pathway selection, potentially delaying access to evidence-based treatment and contributing to disease progression. From a health systems perspective, the findings reflect structural limitations in Ghana’s healthcare financing framework, particularly in relation to cancer care coverage. Although the National Health Insurance Scheme (NHIS) provides some level of support, many cancer-related services, diagnostics, and medications are either partially covered or excluded, resulting in high out-of-pocket payments [70,82]. This contrasts sharply with high-income countries, where comprehensive insurance coverage and subsidized cancer care significantly reduce patient financial burden [83,84].

Moreover, the cost differential between metastatic and non-metastatic disease underscores the economic value of early detection and screening programmes [24]. Investment in early diagnosis not only improves survival outcomes but also reduces long-term healthcare expenditure, a concept widely supported in global oncology economics [68,85]. In the Ghanaian context, however, the absence of structured national screening programmes limits the realization of these benefits [86]. It is also important to consider the indirect costs associated with PCa, including loss of productivity, transportation expenses and caregiver burden, which were not quantified in the study but are known to significantly amplify the overall economic impact [35,36]. These hidden costs further exacerbate the financial strain on households and may contribute to treatment discontinuation. In comparison to other cancers in Ghana, PCa management appears similarly constrained by cost-related barriers, reflecting systemic issues in oncology care delivery. However, given the chronic nature of PCa and the need for long-term management (such as prolonged ADT), the cumulative financial burden may be even greater.

In conclusion, the cost of PCa diagnosis and treatment in Ghana is substantial, variable, and often prohibitive, significantly influencing access to care and treatment decisions. The high cost of managing advanced disease highlights the urgent need for early detection strategies, while the limited affordability and availability of medications call for policy interventions to improve drug pricing and supply chains. Strengthening health insurance coverage, subsidizing cancer care, and improving access to timely risk assessment and diagnostic evaluation may help reduce the economic burden associated with advanced PCa. The cost-effectiveness and feasibility of broader screening strategies in Ghana require dedicated evaluation.

*THEME 5: Barriers and Facilitators to the Acceptance of PCa Screening*

The findings in Table 5 illustrate that prostate cancer (PCa) screening behaviour in Ghana is shaped by a complex interplay of cognitive, sociocultural, economic, and health system factors, with a persistent gap between willingness to screen and actual screening uptake. A consistent observation across the studies is the high expressed willingness to undergo screening under enabling conditions. For instance, 79.2% of respondents indicated willingness to screen if recommended by a healthcare provider [25], while earlier findings (Theme 1) similarly showed near-universal agreement on the benefits of screening [21]. This suggests that attitudinal resistance may not be the primary barrier; rather, structural and informational constraints may limit translation into practice.

One of the most salient facilitators identified is direct recommendation from healthcare professionals, which acts as a strong “cue to action” within the Health Belief Model (HBM) framework [87]. This aligns with global evidence demonstrating that physician endorsement is one of the most powerful predictors of cancer screening uptake, particularly in settings without organized screening programmes [88,89]. However, as noted in Theme 3, limited interaction between men and healthcare providers in Ghana reduces exposure to such cues, thereby constraining screening uptake. Conversely, several barriers to screening were identified, many of which are deeply rooted in knowledge deficits and sociocultural beliefs. A notable issue is the low perceived susceptibility to PCa, with many men not considering themselves at risk [25]. This is consistent with findings from other African settings, where risk perception is often low despite high disease burden, leading to delayed health-seeking behaviour [86,90].

Knowledge gaps also play a critical role. In the study by Maison et al. [26], although 75% of participants had heard of PCa, only 35% understood that it affects men, and a mere 4.3% recognized the role of screening in early detection. Such findings indicate that awareness alone is insufficient to drive preventive action, reinforcing observations from earlier themes. Furthermore, misconceptions such as attributing PCa to spiritual causes (15.6%) highlight the influence of cultural and religious beliefs on health behaviours. Economic barriers are equally significant. The perception that orthodox medical care is expensive (61.7%), coupled with the preference for traditional medicine due to affordability (79.8%), underscores the findings from Theme 4 regarding financial constraints. Cost considerations not only delay screening but also redirect patients toward alternative care pathways, which may not support early detection [72]. Health system-related barriers also emerge, including the lack of organized screening programmes, limited access to diagnostic facilities, and inadequate public health outreach. In contrast to high-income countries where population-based screening and routine check-ups are institutionalized, Ghana relies largely on opportunistic screening, which is inherently less effective [91].

Importantly, psychosocial factors also influence screening behaviour. Although some participants rejected the notion that screening is embarrassing or painful [21], these perceptions remain documented barriers in the broader literature, particularly regarding digital rectal examination (DRE), which may be associated with stigma, discomfort, or perceived threats to masculinity [69,71]. Facilitators identified across studies point toward multi-level intervention opportunities. Community-based awareness initiatives, such as media campaigns, educational talks, flyers and SMS reminders, were suggested as effective strategies to promote screening [21]. These approaches leverage the dominant information channels identified in Theme 3 and could serve as scalable, cost-effective interventions. Additionally, social influence and family history appear to play a role in motivating screening behaviour. Individuals with a known family history of PCa were more likely to engage in discussions and screening practices [25]. This aligns with global evidence that personal relevance and perceived risk significantly enhance preventive health behaviours [68].

From a comparative perspective, barriers identified in Ghana mirror those in other low- and middle-income countries, namely low risk perception, financial constraints, cultural beliefs, and weak health systems [92]. However, the magnitude of these barriers appears amplified in Ghana due to the absence of structured screening programmes and limited health literacy. In contrast, high-income settings benefit from integrated screening frameworks, insurance coverage, and stronger patient–provider communication, which collectively improve screening uptake [75].

Finally, PCa screening in Ghana is hindered by low perceived susceptibility, inadequate knowledge, financial constraints, cultural beliefs, and systemic healthcare limitations, despite high stated willingness to screen. Key facilitators include healthcare provider recommendation, targeted awareness campaigns, and increased risk perception. Addressing these barriers requires a multi-level approach that strengthens primary healthcare engagement, improves community education and risk awareness, reduces financial and geographic barriers to diagnostic assessment, and facilitates timely evaluation of men at increased risk or with suspected disease. The current evidence does not, however, provide a sufficient basis for recommending population-wide PCa screening in Ghana. Any consideration of a national screening programme would require evidence on potential benefits and harms, cost-effectiveness, resource requirements, and the capacity of the healthcare system to provide appropriate diagnostic follow-up and treatment.

*THEME 6: PCa Management and Treatment Outcomes in Ghana*

The evidence presented in Table 6 provides a comprehensive overview of treatment modalities and clinical outcomes of prostate cancer (PCa) in Ghana, revealing a landscape characterized by improving therapeutic capacity within tertiary centres but constrained by late-stage presentation and unequal access to care. The included clinical studies document the use of treatment modalities such as EBRT, brachytherapy, and radical prostatectomy in selected tertiary care settings in Ghana, particularly in Accra. For instance, Mensah et al. [27] reported freedom from biochemical failure rates of 95.4%, 90.9%, and 70.8% for low-, intermediate-, and high-risk patients, respectively, following brachytherapy, outcomes that are broadly comparable to those reported in high-income settings [68,90]. Similarly, Asamoah et al. [28] documented a 5-year overall survival rate of 96% among patients receiving curative radiotherapy, further demonstrating that when patients access appropriate treatment early, outcomes can be highly favourable.

However, these positive outcomes are juxtaposed against a predominance of high-risk and advanced disease at presentation. In the same cohort, 64.4% of patients presented with high-risk disease, while other studies reported elevated prostate-specific antigen (PSA) levels (>20 ng/mL in 69.6% of cases) and substantial proportions of metastatic disease at diagnosis (33.8%) [30]. This pattern is consistent with the broader literature on Sub-Saharan Africa, where late presentation remains a defining feature of PCa epidemiology due to limited screening, poor awareness, and healthcare access barriers [65,66]. Treatment outcomes also vary significantly by disease stage and risk category. While biochemical disease-free survival (bDFS) rates were as high as 95% in low-risk groups, they declined markedly to 48% in high-risk patients [28]. This gradient underscores the critical importance of early detection, as prognosis deteriorates substantially with disease progression. Comparable trends have been observed globally, reinforcing stage at diagnosis as one of the strongest predictors of survival in PCa.

Surgical management, although less commonly utilized, has shown promising outcomes in selected cases. Kyei et al. [31] reported that 95% of the patients maintained PSA levels below 0.4 ng/mL post-prostatectomy, alongside high continence rates and favourable potency recovery (78.6%). However, the high intraoperative blood loss and transfusion rate (70%) reflect potential limitations in surgical infrastructure and perioperative care, which may restrict the widespread adoption of this modality. Radiotherapy remains a cornerstone of PCa management in Ghana. Daniels et al. [29] reported that 86.9% of patients treated with EBRT were free from biochemical progression, indicating strong treatment efficacy. Additionally, improvements in urinary symptoms post-treatment suggest therapeutic benefits beyond disease control. Nevertheless, treatment-related adverse effects, such as the increase in erectile dysfunction from 1.6% to 18.9%, highlight the trade-offs between efficacy and quality of life, a consideration increasingly emphasized in modern oncology [68].

Another notable finding is the predominant reliance on androgen deprivation therapy (ADT), particularly in advanced cases. Mensah et al. [47] reported that 83.9% of patients underwent bilateral orchidectomy, reflecting the use of cost-effective but irreversible treatment options in resource-limited settings. While ADT is effective for disease control, its widespread use, often in the absence of curative interventions, suggests that many patients in these cohorts were managed palliatively rather than curatively, particularly among those presenting with advanced disease. Mortality outcomes further reflect these challenges. Over 50% of patients died within 24 months of diagnosis, although a subset survived beyond 5 years [47]. This variability highlights heterogeneity in disease progression and treatment response, but also underscores the impact of delayed diagnosis and limited access to comprehensive care.

Disease stage was associated with the treatment patterns reported in the reviewed clinical studies, although the available evidence does not establish late presentation as the sole determinant of treatment selection. Asamoah et al. [28] reported that 57.6% of patients with localized disease received curative radiotherapy, while outcomes declined with increasing risk category. Similarly, Yeboah et al. [42] reported different management approaches according to disease extent: localized cases received modalities, including radical prostatectomy, brachytherapy and external beam radiotherapy, whereas locally advanced and metastatic cases were more frequently managed with hormonal therapy. Mensah et al. [47] also reported predominant use of androgen deprivation therapy, including bilateral orchidectomy, among patients who subsequently died from PCa, while only a small proportion had received local curative treatment. These findings indicate an association between disease stage and the type of treatment received, but they should not be interpreted as demonstrating that late presentation alone determines treatment choice.

Other factors identified across the reviewed literature may also shape access to and use of treatment. Wiafe et al. [24] documented substantial treatment costs and limited availability of PCa medications, with mean drug availability ranging from 41.54% to 61.54%, suggesting that affordability and availability may constrain treatment options. Access to specialized treatment is also geographically concentrated in major tertiary centres, while advanced radiotherapy techniques require equipment, planning infrastructure and specialized expertise that may not be uniformly available [32]. In addition, Maison et al. [26] found that 61.7% of participants perceived orthodox medical care as more expensive than traditional care, and some participants reported cultural or religious influences on healthcare choices. These findings suggest that treatment pathways may reflect not only disease characteristics but also financial, geographic, technological and sociocultural circumstances. However, the included studies did not directly investigate patient preferences or clinician decision-making practices as determinants of treatment selection. Future studies should examine these factors explicitly to provide a more complete understanding of how treatment decisions are made in the Ghanaian context.

From a systems perspective, the findings indicate that Ghana has made significant progress in developing oncology treatment capacity, particularly in major urban centres such as Accra. However, access remains geographically and economically uneven, with rural populations facing substantial barriers to specialized care [70]. This inequity contributes to disparities in outcomes and reinforces the need for decentralized cancer services and strengthened referral systems. In comparison to high-income countries, where PCa outcomes have improved due to early detection, advanced technologies, and multimodal treatment approaches [93], Ghana’s outcomes are constrained primarily by late presentation and resource limitations rather than lack of therapeutic efficacy [4,42]. Indeed, when comparable treatments are applied, clinical outcomes in Ghanaian cohorts are largely consistent with global benchmarks, suggesting that the primary gap lies in access and timing of care rather than treatment capability.

Overall, PCa management in selected tertiary care studies in Ghana reported favourable outcomes among some patients with localized disease, supported by the availability of radiotherapy, brachytherapy, and surgical interventions. However, the high prevalence of advanced-stage presentation, reliance on palliative therapies, and disparities in access to care significantly limit overall survival outcomes. Strengthening early detection strategies, expanding access to curative treatments, and improving healthcare infrastructure are essential to optimizing PCa outcomes in Ghana.

*THEME 7: Dosimetric Comparison of Radiotherapy Techniques in PCa*

The findings in Table 7 focus on the comparative efficacy of radiotherapy techniques in prostate cancer (PCa) management, particularly within the context of a resource-limited setting such as Ghana. The study by Kyei et al. [32] provides critical insight into the dosimetric advantages of intensity-modulated radiotherapy (IMRT) over three-dimensional conformal radiotherapy (3DCRT), highlighting important implications for treatment optimization and patient outcomes. A central finding is that IMRT demonstrated better dose conformity and homogeneity compared to 3DCRT, with significantly improved sparing of organs at risk (OARs). Specifically, the mean homogeneity indices were markedly lower in IMRT (0.01–0.011) compared to 3DCRT (0.04–0.016). Additionally, IMRT achieved more precise dose distribution, minimizing radiation exposure to surrounding critical structures such as the bladder and rectum. These findings are consistent with global evidence, where IMRT is widely regarded as the standard of care in modern radiotherapy for localized PCa due to its ability to deliver high radiation doses to tumours while reducing toxicity [94,95].

The clinical significance of these dosimetric improvements lies in their direct impact on treatment-related morbidity and quality of life [96]. Reduced radiation exposure to adjacent organs is associated with lower rates of gastrointestinal and genitourinary toxicities, which are common complications of prostate radiotherapy [75]. This is particularly relevant in the Ghanaian context, where treatment-related side effects can significantly affect adherence and long-term outcomes, especially in the absence of supportive care systems. Despite the demonstrated superiority of IMRT, its implementation in Ghana and similar settings is often constrained by resource limitations, including high equipment costs, the need for advanced planning systems, and specialized technical expertise. Consequently, 3DCRT remains widely used due to its relative affordability and availability, even though it may offer a less optimal dose distribution. This reflects a broader challenge in oncology care across low- and middle-income countries, where treatment decisions are frequently influenced by resource availability rather than clinical superiority [96].

Importantly, the study demonstrates that technological advancements in cancer treatment are feasible within the Ghanaian healthcare system, particularly in tertiary centres. The successful application of IMRT indicates growing capacity for advanced oncologic care, which could significantly improve patient outcomes if scaled appropriately. However, equitable access remains a concern, as such technologies are typically concentrated in urban centres, limiting availability to a subset of the population [70]. When contextualized within the broader treatment outcomes discussed in Theme 6, the superiority of IMRT suggests that further improvements in survival and quality of life could be achieved through wider adoption of advanced radiotherapy techniques [97,98]. However, these benefits are contingent upon early diagnosis, as advanced-stage disease may limit the applicability of localized radiotherapy altogether.

Comparatively, in high-income countries, IMRT has largely replaced older radiotherapy techniques, supported by robust infrastructure, trained personnel, and health insurance systems that cover advanced treatments [99]. In contrast, Ghana’s reliance on both IMRT and 3DCRT reflects a transitional phase in oncology service delivery, where modernization is occurring but not yet universal [100]. Another important consideration is cost-effectiveness. While IMRT offers superior clinical outcomes, its higher upfront costs may pose barriers in low-resource settings. However, evidence suggests that IMRT may be cost-effective in the long term due to reduced complication rates and associated healthcare costs [101]. This highlights the need for economic evaluations tailored to the Ghanaian context to inform policy decisions regarding investment in radiotherapy infrastructure.

In conclusion, the findings from this theme reported better dose conformity, homogeneity, and organ-at-risk sparing with IMRT than with 3DCRT. These dosimetric advantages support the potential value of IMRT in treatment planning, but this study did not directly evaluate survival, quality of life, or long-term clinical toxicity. Further clinical and economic evaluations are therefore required before conclusions can be drawn regarding comparative patient outcomes in Ghana. Expanding access to advanced radiotherapy technologies, alongside investments in training and infrastructure, could be critical for improving PCa treatment outcomes in Ghana.

*THEME 8: Psychosocial Impact on People Living with Prostate Cancer*

The studies presented in Table 8 collectively demonstrate that prostate cancer in Ghana exerts a profound multidimensional psychosocial burden, affecting patients’ identity, sexual function, emotional wellbeing, and overall quality of life. The qualitative study by Salifu et al. [33] provides in-depth insight into how advanced prostate cancer disrupts masculine identity, a culturally significant construct in the Ghanaian context. Over half of the men reported diminished masculinity, driven by changes in physical appearance, reduced sexual performance, and altered socioeconomic roles. These disruptions were strongly associated with emotions of fear, weakness, and loss of self-worth, with caregivers corroborating these experiences. This finding underscores the centrality of masculinity in shaping illness perception and psychological response among men with prostate cancer.

Similarly, Daniels et al. [23] highlight the significant impact of both the disease and its treatment on sexual function, with patients reporting reduced sexual desire (54%), premature ejaculation (49%), and decreased sexual activity (48%). Radiotherapy further contributed to erectile dysfunction (49%) and additional declines in sexual desire and performance, reinforcing the compounding effect of treatment-related side effects on psychosocial wellbeing. These findings illustrate that sexual dysfunction is not only a physical consequence but also a key driver of psychological distress and diminished quality of life. Expanding on overall wellbeing, Ntiamoah and Senoo-Dogbey [12] reported low health-related quality of life (HRQoL) among patients, with a mean FACT-P score of 72. Emotional wellbeing was significantly poorer among treated patients, while factors such as advanced age, erectile dysfunction, bowel problems, and anxiety were identified as predictors of worse outcomes. Although treated patients showed slightly better social and functional wellbeing, the overall findings indicate a persistent psychosocial burden regardless of treatment status.

Further reinforcing these findings, Annan et al. [34] documented substantial psychological distress among unmarried men, including anxiety, depression, distress, and even suicidal ideation. These symptoms were linked to pain, urinary complications, erectile dysfunction, and financial strain, highlighting the interplay between physical symptoms and mental health. Coping strategies such as lifestyle modification, social interaction, and acceptance were reported, though these varied in effectiveness.

Across studies, a consistent pattern emerges: prostate cancer significantly affects psychological wellbeing, sexual health, identity, and social functioning, with these impacts often intensified by treatment side effects and socioeconomic challenges. These findings align with the global literature indicating that prostate cancer is associated with substantial psychosocial morbidity, particularly in contexts where cultural expectations around masculinity and limited psychosocial support services exacerbate patient experiences.

*THEME 9: Psychosocial Impact on Caregivers of Persons Living with Prostate Cancer*

The findings from Table 9 reveal that caregiving for individuals with prostate cancer in Ghana is associated with significant emotional, physical, social, and financial burden, often occurring in resource-constrained settings with limited institutional support. The phenomenological study by Ninnoni and Owoo [35] highlights the emotional strain experienced by caregivers, characterized by anxiety related to financial constraints, changes in patients’ physical condition, and the uncertainty surrounding cancer diagnosis and treatment. Caregivers also reported role disruption, reduced leisure time, and diminished quality of sexual life, reflecting the pervasive impact of caregiving responsibilities on personal wellbeing.

Similarly, Appiah et al. [36] found that spouses of men with prostate cancer experienced considerable physical and emotional stress, including sleeplessness, exhaustion, decreased appetite, and persistent anxiety. Many participants expressed fear of losing their partners and distress related to medical procedures and the potential long-term consequences of the disease, such as infertility and reduced sexual function. These findings emphasize the emotional toll of anticipatory grief and uncertainty in caregiving. The studies by Owoo et al. [37,39] further illuminate the system-level challenges faced by caregivers. Participants reported inadequate knowledge about the disease, leading to confusion and panic, as well as financial difficulties associated with treatment and transportation costs. In addition, caregivers encountered institutional barriers, including inadequate hospital facilities and limited support from healthcare professionals. Physically, caregiving was associated with sleep deprivation, pain, altered dietary patterns, and weight loss, indicating substantial physical strain.

In a related study, Salifu et al. [38] highlighted the challenges of home-based care in resource-poor settings, where both patients and caregivers reported limited access to healthcare support and medications. Caregivers often assumed extended roles, effectively acting as informal healthcare providers without adequate training, leading to increased burden and stress. Across these studies, several consistent themes emerge: high emotional burden, including anxiety, fear, and distress; significant physical strain, including fatigue, pain, and sleep disturbances; financial challenges, driven by treatment costs and limited resources; and lack of adequate support systems, both within healthcare settings and at home. These findings suggest that caregiving in the context of prostate cancer in Ghana is largely informal, unsupported, and resource-intensive, placing caregivers at risk of poor health outcomes. This aligns with broader evidence from low- and middle-income countries, where caregiving responsibilities are often transferred to family members without adequate institutional support, leading to caregiver burden and reduced quality of life.

Importantly, the findings highlight opportunities for intervention. Integrating psychosocial care into PCa management, including counselling services, patient education, and peer support groups, could significantly improve quality of life [102]. Community-based interventions that address stigma and misconceptions may also enhance social support and encourage earlier health-seeking behaviour. Taken together, Table 8 and Table 9 highlight that prostate cancer in Ghana extends beyond a clinical condition to become a shared psychosocial experience affecting both patients and caregivers. The disease disrupts identity, relationships, emotional wellbeing, and economic stability, with limited access to psychosocial and institutional support systems exacerbating these effects.

*THEME 10: Risk Factors of PCa*

The evidence summarized in Table 10 includes established non-modifiable risk factors for prostate cancer (PCa), particularly increasing age, family history, genetic susceptibility, and African ancestry, as well as several lifestyle, metabolic, and environmental exposures that have been investigated for possible associations with the disease. A consistent and well-established determinant across the included studies is advancing age, with the majority of cases occurring in men aged ≥50 years [42,70]. This aligns with global epidemiological evidence identifying age as the strongest risk factor for PCa, largely due to cumulative cellular mutations, prolonged androgen exposure, and age-related changes in immune surveillance [68]. In the Ghanaian context, increasing life expectancy may therefore contribute to the rising incidence of PCa observed in recent years [4].

Another prominent non-modifiable risk factor is genetic predisposition and African ancestry [103]. Men of African descent, including Ghanaians, have been shown to have a higher incidence, earlier onset, and more aggressive forms of PCa compared to other populations [26,104]. Although the specific genetic mechanisms remain under investigation, polymorphisms in genes related to androgen metabolism and tumour suppression have been implicated [105]. Family history also emerges as an important risk factor, with individuals having a first-degree relative diagnosed with PCa exhibiting significantly increased risk [25]. However, awareness of familial risk appears limited among Ghanaian men, potentially reducing opportunities for targeted screening and early intervention in high-risk groups [86].

Several potentially modifiable exposures have also been investigated, although they should not be interpreted as established avoidable causes of PCa. Dietary patterns, including high intake of saturated fats, red meat, and processed foods and Western-style diets, have been associated with PCa in some observational and meta-analytic studies [106,107]. Similarly, obesity and measures of adiposity have been associated with PCa risk or disease characteristics. In the Ghana Prostate Study, Hurwitz et al. [45] reported associations between obesity, waist circumference, waist-to-hip ratio, and PCa. However, these associations do not establish that modifying body weight or diet will prevent PCa. Evidence for physical activity and lifestyle exposures is likewise insufficient to establish these factors as validated preventable causes of the disease [68,103]. These factors should therefore be interpreted as possible correlates or areas for further investigation rather than confirmed targets for PCa prevention. Although these exposures have been investigated in the broader literature, their specific relevance to PCa development among Ghanaian men requires further study. Environmental exposures, including potential contact with agrochemicals and industrial pollutants, have also been suggested as risk factors, although evidence in the Ghanaian context is still emerging [108]. These exposures may contribute to carcinogenesis through endocrine disruption and oxidative stress, but further research is needed to establish causal relationships.

An important observation from the reviewed studies is the limited public awareness of PCa risk factors, as highlighted in Theme 1. For example, a substantial proportion of participants were unable to identify known risk factors, while some attributed the disease to incorrect causes such as mobile phone radiation or spiritual factors [21]. This gap in knowledge has significant implications, as risk perception is a key driver of preventive health behaviour, including screening uptake [25]. In high-income countries, greater awareness of risk factors supports risk-stratified screening approaches, enabling earlier detection among high-risk populations [109]. From a public health perspective, the identification of both modifiable and non-modifiable risk factors presents an opportunity for targeted interventions. While non-modifiable factors such as age and genetics cannot be altered, they can inform risk stratification and screening guidelines [2]. Although healthy diets, regular physical activity, and maintenance of a healthy body weight have important general health benefits, the available evidence does not establish these behaviours as specific preventive measures against PCa. Their promotion should therefore be framed primarily within broader chronic disease prevention rather than as proven PCa prevention strategies.

In conclusion, the most clearly established PCa risk factors relevant to Ghanaian men are largely non-modifiable, including increasing age, family history, genetic susceptibility, and African ancestry. Lifestyle and metabolic factors such as diet and adiposity have been associated with PCa in some observational studies, but these relationships remain insufficient to define them as validated avoidable risk factors. Future Ghana-specific research should clarify these associations while prevention messages distinguish established PCa risk factors from general healthy-lifestyle recommendations.

*THEME 11: Biomarkers and Diagnostic Approaches of PCa in Ghana*

The reviewed evidence from Table 11 indicates that prostate-specific antigen (PSA) was the most frequently investigated biomarker within the identified screening, diagnostic, and staging-related studies, although it was rarely assessed in isolation. Seven studies directly evaluated PSA within screening, diagnostic, or staging/prediction pathways [43,46,50,51,52,53,54]. Several of these studies combined PSA with other clinical or diagnostic measures, particularly digital rectal examination (DRE), prostate biopsy, histopathological assessment, and additional biomarkers. DRE was assessed alongside PSA in five studies [43,46,50,51,54]. Mensah et al. [46], for example, analysed 7338 men who underwent transrectal ultrasound-guided prostate biopsy and found that increasing PSA levels and higher DRE stages corresponded with a greater probability of cancer detection. Similarly, Yeboah et al. [50] incorporated PSA, PSA density, DRE, age, smoking history, and alcohol consumption into a nomogram for predicting prostate biopsy outcomes. In the population-based screening study by Hsing et al. [54], 352 of 1037 men screened positive by PSA or DRE, 307 underwent biopsy, and 73 were confirmed to have PCa, corresponding to a screen-detected prevalence of 7%. These findings show that PSA was commonly used as part of a broader diagnostic pathway rather than as a standalone test. Likewise, Wiredu and Armah [110], in their histopathological review of PCa in Ghana, reported that diagnosis was typically confirmed following abnormal PSA findings and subsequent tissue biopsy, underscoring the integration of PSA testing within diagnostic pathways.

Biopsy and histopathological assessment were also important for confirming disease and characterizing tumour features. Biopsy or histopathological confirmation was explicitly incorporated in five studies [43,46,50,53,54]. Histopathological grading using the Gleason score provided additional information on tumour aggressiveness. Yarney et al. [40] reported that 56% of patients had Gleason scores ≥7, indicating a substantial proportion of intermediate- to high-grade disease, while Bosson-Amedenu et al. [41] reported a mean Gleason score of 7.47 among men diagnosed with PCa. Gleason grading was also reported in treatment-related studies; Kyei et al. [31], for example, reported a mean Gleason score of 6.5 before surgery and 6.8 following pathological assessment. Thus, Gleason scoring complemented PSA, DRE, and histopathological confirmation by providing information on tumour grade and aggressiveness relevant to clinical risk stratification.

Imaging was less prominently represented in the reviewed diagnostic literature. Although multiparametric magnetic resonance imaging (mpMRI) is increasingly incorporated into PCa diagnostic pathways internationally, none of the eligible Ghanaian studies specifically evaluated its availability or use. Therefore, the present review cannot determine the extent to which mpMRI is incorporated into routine PCa diagnosis in Ghana, highlighting an important area for future health-services and diagnostic research. Obeng et al. [43] evaluated treatment-naïve patients undergoing staging bone scans and examined PSA together with alkaline phosphatase (ALP), DRE stage, and histopathological characteristics for the prediction of bone metastasis. The study showed that the addition of ALP, International Society of Urological Pathology (ISUP) grade, and DRE stage improved the ability of PSA to identify bone metastasis. This suggests potential value in combining biochemical, clinical, histological, and imaging information when assessing disease extent.

A smaller body of research has explored alternative and adjunctive biomarkers for PCa assessment in Ghana. Opoku Mensah et al. [51] evaluated urinary PCA3 and reported better diagnostic accuracy than PSA or DRE when each was considered individually, with the combination of PCA3 and DRE providing the best diagnostic performance. Appiah et al. [52] investigated serum ferritin and hepcidin as complementary biomarkers and found significantly higher concentrations among men with PCa than among those with benign prostatic enlargement or healthy controls. In addition, Obeng et al. [43] assessed serum alkaline phosphatase (ALP) as an adjunct to PSA for predicting bone metastasis, while Yeboah et al. [50] incorporated PSA density into a multivariable model for predicting prostate biopsy outcomes.

These findings indicate potential diagnostic or predictive value but should be interpreted as preliminary rather than evidence of established clinical implementation. The included studies do not demonstrate that PCA3, ferritin, or hepcidin are routinely used in PCa diagnosis in Ghana, and further prospective validation is required to establish their reproducibility, clinically useful thresholds, and performance in routine care. Similarly, genomic studies examining TMPRSS2 fusion, single nucleotide polymorphisms, and long non-coding RNAs provide important insight into the molecular characteristics of PCa among Ghanaian men, but these markers remain primarily research tools rather than established components of routine clinical diagnosis. In contrast, PSA, DRE, biopsy, histopathological assessment, and Gleason grading are more consistently represented across the clinical studies reviewed. This distinction is important when interpreting emerging biomarker research, as promising experimental or diagnostic performance does not necessarily indicate clinical validation or routine use.

The limitations of PSA alone are further reflected in studies evaluating complementary approaches. Opoku Mensah et al. [51] reported greater diagnostic accuracy for urinary PCA3 than for PSA or DRE individually, while Obeng et al. [53] found that a multiparametric model outperformed a PSA-only model in predicting metastasis. Thus, PSA is better interpreted as part of a broader assessment pathway incorporating clinical examination, risk factors, biopsy, histopathology, and, where appropriate, additional diagnostic tools. Although improving access to timely PSA testing may contribute to earlier detection in selected men, wider screening strategies in Ghana should also consider the potential for unnecessary investigations, diagnostic capacity, affordability, and the balance of benefits and harms. The reviewed literature did not assess whether PSA assays performed across public and private laboratories followed comparable analytical quality-control procedures; consequently, the consistency of PSA measurement across settings could not be determined.

Overall, PSA remains a prominent component of PCa assessment in the Ghanaian evidence base, but the reviewed studies demonstrate that diagnostic evaluation is multimodal rather than exclusively PSA-based. This is because variability in PSA levels across patients and overlap with benign prostatic conditions indicate that PSA lacks sufficient specificity as a standalone diagnostic tool, necessitating histological confirmation [111]. DRE, biopsy, histopathological assessment, and Gleason grading are important components of disease detection and characterization, while imaging and newer candidate biomarkers have received comparatively less investigation. Further prospective studies are needed to validate emerging biomarkers and multimodal diagnostic approaches in Ghanaian populations before their broader clinical application.

*THEME 12: Genetic and Genomic Characteristics of PCa in Ghana*

Table 12 presents emerging evidence on the molecular and genetic characteristics of PCa relevant to Ghanaian populations. However, the evidence is based on a small number of studies, and some studies included broader African-ancestry populations; therefore, Ghana-specific findings should be distinguished from findings generated from mixed ancestry samples. The study by Zhou et al. [55] provides important insight into *ERG gene* expression, specifically examining the presence of *TMPRSS2:ERG* gene fusion, a well-established molecular alteration in PCa. The paper investigated *ERG* expression in prostate cancer specimens from the Ghana Prostate Study, which enrolled 1037 Ghanaian men aged 50–74 years, with *ERG* staining assessed in 262 prostate cancer biopsies. The findings indicate that negative *ERG* staining was associated with higher Gleason scores, suggesting more aggressive disease in patients lacking this fusion. Notably, the study also reported a lower prevalence of *TMPRSS2:ERG* fusion in the Ghanaian samples. This finding suggests that the frequency of this molecular alteration may differ from that reported in some other ancestry groups, but further Ghana-specific genomic studies are required to determine the biological and clinical significance of this observation.

Similarly, Chung et al. [56] employed next-generation sequencing to investigate genetic variation in 125 individuals of African ancestry, including 78 Ghanaian participants. The study identified genetic variants across the broader African-ancestry sample, including 135 novel single nucleotide polymorphisms (SNPs) reported specifically in the Ghanaian samples, as well as three long non-coding RNAs (lncRNAs) within the targeted genomic region. The Ghana-specific SNP findings are relevant to understanding genetic variation among Ghanaian participants; however, because the overall study population was not exclusively Ghanaian, findings derived from the broader sample should not be interpreted as population-specific characteristics of Ghanaian men. The study nevertheless highlights the importance of including African and Ghanaian populations in genomic research, given their historical underrepresentation in global genomic datasets. Further supporting the role of genetic susceptibility, Cook et al. [57] investigated genetic susceptibility among 500 Ghanaian men with prostate cancer. The study identified SNPs at chromosomal region 5q31.3 associated with higher Gleason scores (≥7). These findings suggest an association between specific genetic variants and tumour grade in the study population, but they do not establish a causal relationship between these variants and disease aggressiveness. Further independent studies are needed to confirm the observed associations.

Taken together, the available studies provide preliminary evidence of genetic and molecular characteristics that may be relevant to PCa in Ghanaian populations. These include a relatively low prevalence of *TMPRSS2:ERG* fusion in the Ghanaian samples studied, novel SNPs reported specifically among Ghanaian participants within a broader African-ancestry study, and genetic variants associated with tumour grade. [112,113]. However, the evidence is based on a small number of studies, some involving broader African-ancestry populations, and independent replication remains limited. It is therefore premature to define a distinct Ghanaian genomic profile of PCa. A key implication of these findings is the limitation of generalizing genomic data from non-African populations to African settings [114]. Genomic risk-prediction models and precision medicine approaches developed largely from European-ancestry datasets may not fully capture the genetic diversity represented in Ghanaian populations, which may affect their transferability and predictive performance [115]. Despite these important contributions of the available Ghanaian studies, substantial gaps in genomic research remain. The number of studies is small, and much of the evidence is exploratory, with considerably smaller samples than those available in large international genomic consortia [116]. This underscores the need for larger, Ghana-specific and multicentre genomic studies, independent validation of identified variants, and greater inclusion of Ghanaian populations in genomic research. Where clinically relevant associations are confirmed, future research should also evaluate their potential application to locally appropriate risk prediction, biomarkers, and precision approaches to PCa care.

Although the studies summarized in Table 12 provide important insights into the molecular and genetic characteristics of prostate cancer relevant to Ghanaian populations, the evidence remains exploratory. The reviewed studies differed in sample size, genomic methods, and study populations, and the reported genomic findings were not independently replicated or externally validated in separate Ghanaian cohorts among the studies included in this scoping review. Larger genomic studies with independent validation cohorts are therefore needed to determine the reproducibility, clinical relevance, and potential utility of these findings for risk prediction, diagnosis, prognosis or treatment selection.

*THEME 13: Dietary and Metabolic Factors Relevant to PCa*

Evidence on dietary and metabolic factors relevant to prostate cancer (PCa) in Ghana was limited. Following application of the eligibility criteria, only one study in Table 13 directly examined a dietary exposure in relation to metabolic and prostate-related markers. Ntim et al. [58] conducted an analytical cross-sectional study among 415 Ghanaian men aged 40 years and above in the Ashanti Region to examine associations between green tea consumption, body mass index (BMI), serum lipid profile, and serum prostate-specific antigen (PSA). Green tea drinkers had more favourable BMI and lipid profiles than non-tea drinkers, including lower levels of atherogenic lipids. Lower mean serum PSA levels were also observed among green tea drinkers in the older age groups (60–69 and ≥70 years), whereas this difference was not observed among younger participants [58].

These findings suggest a possible relationship between green tea consumption and metabolic and prostate-related biomarkers, but they should be interpreted cautiously. The cross-sectional design does not establish that green tea consumption caused the observed differences in BMI, lipid profile, or PSA, nor does a lower serum PSA level demonstrate a reduction in PCa incidence or risk. The study therefore provides preliminary evidence of associations with selected metabolic and prostate-related indicators rather than evidence that green tea prevents PCa. This distinction is particularly important because PSA is a biomarker used in prostate assessment and can be influenced by factors other than malignancy. The potential relationship between green tea and PCa has also been examined internationally, with mixed findings. A systematic review and meta-analysis by Guo et al. [117] found no statistically significant difference in PCa risk when the highest and lowest categories of green tea consumption were directly compared, although a dose-response analysis suggested a possible reduction in risk at higher levels of consumption. Green tea catechins have similarly attracted interest because of their biological activity, but the authors emphasized the need for further studies to confirm their preventive effects [117]. These findings support cautious interpretation of the Ghanaian results rather than assuming a protective effect of green tea.

The metabolic findings reported by Ntim et al. [58] are also relevant in the context of evidence linking adiposity to PCa. Within the Ghanaian literature included in this review, Hurwitz et al. [45] found that obesity, elevated waist circumference, and higher waist-to-hip ratio were associated with increased odds of PCa in the Ghana Prostate Study. Broader evidence also suggests an association between elevated BMI and PCa risk. A recent systematic review and meta-analysis reported higher PCa risk among overweight and obese men compared with those of normal weight, although heterogeneity across studies indicates that the relationship is complex [118]. These findings provide context for investigating metabolic health in Ghanaian PCa research but do not establish that the BMI or lipid differences observed among green tea drinkers directly alter PCa risk.

Overall, Ghana-specific evidence on nutrition and PCa remains sparse. The available study suggests associations between green tea consumption and favourable BMI, lipid profiles, and lower PSA levels among some older Ghanaian men, but it does not establish a protective effect against PCa [58]. Important areas remain largely unexplored, including habitual dietary patterns, nutrient intake, food processing, obesity-related metabolic factors, nutritional status after diagnosis, and the influence of diet on treatment outcomes and survivorship. Future studies should therefore use prospective and appropriately controlled designs to determine whether specific dietary and metabolic factors are associated with PCa incidence, progression, or clinical outcomes in Ghanaian men.

*THEME 14: Pharmacological Interventions in PCa Management*

The findings from Table 14 highlight emerging pharmacological research in Ghana focused on natural products with potential antiprostate cancer activity, primarily evaluated through in vitro experimental models. The study by Tettey et al. [59] investigated the aqueous root extract of *Croton membranaceus* (Vikil^20^) for its antioxidative and antiprostate cancer properties. The findings demonstrated strong antioxidant activity, with suppression of 57.61% and 92.88% of DPPH and ABTS radicals, respectively. In addition, the extract exhibited significant cytotoxic effects on PC-3 PCa cells, reducing cell viability to 49.5%, and markedly inhibiting cell migration and adhesion within 48 h. These results suggest that *Croton membranaceus* may exert anti-cancer effects through both oxidative stress modulation and inhibition of tumour cell proliferation and metastatic potential. Similarly, Adotey et al. [60] evaluated the cytotoxic activity of mycelial biomass extracts of *Ganoderma weberianum* isolated from the Lower Volta River Basin. Their findings showed that fractions of the extract significantly inhibited the proliferation and survival of PCa (PC-3) cells in a dose-dependent manner, indicating potent antiproliferative effects. The study further demonstrated activity across multiple cancer cell lines, suggesting broad-spectrum cytotoxic potential.

These findings indicate a growing interest in ethnopharmacology and natural product-based drug discovery in the Ghanaian context [119]. Importantly, both studies utilize the PC-3 cell line, a well-established model for androgen-independent (advanced) PCa, suggesting relevance to aggressive disease phenotypes commonly observed in Ghana [120]. However, despite these promising findings, the evidence remains preclinical and exploratory. The absence of in vivo studies, clinical trials and pharmacokinetic evaluations limits the direct applicability of these agents in clinical practice. Additionally, issues such as dosage standardization, toxicity profiles, and bioavailability remain unaddressed [121]. In relation to the broader literature, these findings align with global research trends exploring plant-derived compounds and medicinal fungi as potential anti-cancer agents, particularly due to their antioxidant, anti-inflammatory, and antiproliferative properties [122]. For instance, species within the *Ganoderma* genus have been widely studied for their immunomodulatory and anti-tumour effects, while plant-based compounds are increasingly investigated as adjuncts or alternatives to conventional chemotherapy [123]. Nonetheless, compared to high-income settings where pharmacological management includes androgen receptor pathway inhibitors, chemotherapy, and targeted therapies, the Ghanaian evidence base remains largely experimental and not yet translated into clinical application [124]. This highlights a critical gap between basic research and clinical implementation.

Overall, Table 14 demonstrates that pharmacological research on PCa in Ghana is currently centred on experimental natural product studies, with promising in vitro evidence of antiproliferative and anti-metastatic effects. However, the lack of clinical validation underscores the need for progression from laboratory studies to in vivo and clinical trials to determine safety, efficacy, and therapeutic potential. Further preclinical and clinical evaluation would be required before these agents could be considered for clinical integration.

*THEME 15: Epidemiological Measures of PCa in Ghana*

The findings from Table 15 provide different measures of the burden of prostate cancer in Ghana, including hospital-based case proportions, population-based incidence, and screen-detected prevalence. These measures have different denominators and study populations and should therefore not be interpreted as directly comparable estimates of population-level prevalence. The hospital-based registry study by Calys-Tagoe et al. [61], conducted at a major tertiary referral centre in Accra, reported an overall cancer prevalence of 274.7 per 100,000 hospital attendees, with males accounting for 29.8% of cases. Notably, PCa represented 26.5% of male cancer cases recorded in the hospital registry, indicating that it is one of the leading malignancies among men in tertiary care settings. This represents the proportion of cancers among male patients captured by a tertiary hospital registry rather than the prevalence of prostate cancer in the Ghanaian male population. This high proportion likely reflects both true disease burden and referral bias, as Korle Bu Teaching Hospital serves as a national referral centre. Similarly, Adadey et al. [63], in a retrospective hospital-based study in the Volta Region, reported that PCa accounted for 16.9% of diagnosed cancer cases identified from hospitals in the region between 2012 and 2014, further supporting its prominence among male cancers across different regions. This percentage is also a case proportion rather than a prostate cancer incidence rate or population prevalence estimate. However, the study also reported relatively low overall cancer incidence rates (approximately 9–11 per 100,000 population across years), suggesting potential underdiagnosis or underreporting, particularly in less resourced settings.

In contrast, population-based data from Amoako et al. [62], using the Kumasi Cancer Registry, provided a different perspective. The study reported a cancer incidence of 37.1 per 100,000 among men, with PCa specifically accounting for 10.5 per 100,000 men in 2015. Compared to hospital-based findings, these figures are lower, reflecting the more representative nature of population-based cancer registries, which capture cases beyond tertiary referral centres. Further insight into the underlying burden is provided by Hsing et al. [54], who conducted different epidemiological measures through a population-based screening study among healthy men aged 50–74 years in Accra. The study screened 1037 men using PSA and DRE, where 73 participants were confirmed to have prostate cancer following biopsy, yielding a screen-detected prevalence of 7.0%. Importantly, the available epidemiological evidence therefore cannot be summarized as a single national prevalence estimate.

Hospital-based studies tend to overrepresent severe and referred cases, while population-based registries provide more conservative but generalizable estimates [125]. Screening studies, on the other hand, reveal the hidden burden of asymptomatic disease, which is particularly important in settings with low screening uptake. In relation to the broader literature, the findings are consistent with evidence from Sub-Saharan Africa, where PCa is among the most commonly diagnosed cancers in men, but is often underreported due to limited screening and weak cancer surveillance systems [81,126]. Compared to high-income countries, Ghana exhibits lower reported incidence but a higher likelihood of late-stage diagnosis, reflecting disparities in early detection and healthcare access [75]. These differences in study design, denominator, geographic coverage, and participant selection limit direct comparison across studies. More comprehensive population-based cancer registration across Ghana would strengthen estimates of prostate cancer incidence and geographic variation, while appropriately designed population studies would be required to establish national prevalence. Contributing factors include limited access to diagnostic services, low screening uptake and incomplete cancer registry coverage, particularly in rural areas [127].

In conclusion, the available studies in Table 15 provide useful but heterogeneous evidence on the burden of prostate cancer in Ghana. Hospital-based studies report case proportions within selected clinical populations, while population-based registry data provide incidence estimates and screening studies provide screen-detected prevalence within defined groups. These measures should not be interpreted interchangeably or extrapolated to the national population. The current evidence therefore highlights the need for broader population-based cancer registration and nationally representative studies to generate more reliable estimates of prostate cancer incidence, prevalence, and geographic variation in Ghana.

### 4.1. Socioeconomic and Healthcare System Barriers to PCa Care

The evidence synthesized in this review indicates that access to prostate cancer (PCa) detection and treatment in Ghana is shaped not only by disease characteristics but also by socioeconomic and healthcare system factors. Affordability is one of the clearest barriers identified in the reviewed literature. Wiafe et al. [24] reported substantial costs associated with diagnosis and treatment, together with limited availability of some PCa medications. Similarly, Maison et al. [26] found that 61.7% of participants perceived orthodox medical care as more expensive than traditional care. These findings suggest that financial constraints may influence when and where men seek care and may also affect their ability to complete diagnostic assessment or treatment.

The financial burden of PCa care should also be considered within the context of health insurance coverage and household income in Ghana. The National Health Insurance Scheme (NHIS) is intended to reduce out-of-pocket healthcare expenditure, and prostate cancer care was announced for inclusion in its benefit package from 2023 [128]. Nevertheless, insurance coverage does not necessarily eliminate expenses associated with diagnostic investigations, medicines, transportation, repeated hospital visits and other components of care. This is particularly relevant given substantial income disparities across the country. Based on 2023 income estimates, average monthly income was approximately US$134 nationally, compared with about US$158 in the Ashanti Region and US$86 in the Northern Region [129]. Against this background, the costs reported by Wiafe et al. [24] for PCa diagnosis and treatment may represent a considerable proportion of household income, particularly among lower-income populations. Income inequality may therefore compound geographic and health system barriers to timely diagnosis and continuity of treatment.

Access is further influenced by the concentration of specialist cancer services in major urban centres. Much of the clinical evidence in this scoping review originated from tertiary facilities in Accra and Kumasi, where specialized diagnostic and treatment services are more readily represented. Advanced treatment modalities such as intensity-modulated radiotherapy require specialized equipment, planning systems, and technical expertise [31], which may limit their availability outside major referral centres. For patients living farther from these facilities, the costs of transportation, accommodation, repeated hospital visits, and time away from work may add to the direct financial burden of treatment.

Diagnostic capacity is another important consideration. PSA testing, DRE, biopsy, histopathology and some imaging approaches were reported in the reviewed studies; however, the literature did not really assess the national availability, affordability, waiting times or capacity of pathology and imaging services. Likewise, the geographic distribution of urologists, oncologists, pathologists, radiologists and other specialist personnel involved in PCa care was not directly evaluated. These issues therefore remain important health system research gaps rather than established deficiencies within the current evidence base.

Overall, the available evidence indicates that affordability, treatment availability, infrastructure, and geographic location may influence access to PCa care in Ghana. Further research is needed to map specialist workforce capacity and the distribution of diagnostic and treatment services, and to determine how these factors affect timely and equitable care across urban and rural populations.

### 4.2. Limitations

This scoping review has several limitations that should be considered when interpreting its findings. First, although a comprehensive search strategy was implemented across four major electronic databases, the review was restricted to studies published in English and to peer-reviewed journal articles. Consequently, relevant evidence contained in grey literature, conference proceedings, dissertations, government reports, theses, or publications in other languages may have been missed. Relevant evidence may therefore have been omitted, particularly research conducted in institutions or geographic areas with limited publication in indexed journals. This restriction may contribute to both publication bias and the apparent geographic concentration of the available evidence in major urban and academic centres. Second, the review was designed to map the breadth of available evidence rather than quantitatively synthesize findings. Therefore, no meta-analysis was performed, and variations in study populations, methodologies, outcome measures, and reporting practices precluded direct comparison of findings across studies.

Third, the evidence base identified was dominated by observational, hospital-based studies conducted primarily in tertiary healthcare facilities. Because most included studies were observational, and the purpose of this scoping review was to map rather than establish causal relationships, associations reported in the literature should not be interpreted as evidence of causality. This concentration limits the representativeness of the available literature, particularly for rural populations and regions where specialized oncology services are limited. The geographical clustering of studies within the Greater Accra and Ashanti Regions further suggests that important regional differences in prostate cancer awareness, screening practices, diagnosis, treatment access, and survivorship may remain underrepresented. Consequently, the geographic gaps identified should be interpreted as gaps in the published evidence base rather than definitive evidence that no prostate cancer-related work has been undertaken in those regions.

The search was restricted to PubMed, Scopus, ScienceDirect, and the Cochrane Library, which were selected for their complementary coverage of biomedical, clinical, public health, and multidisciplinary peer-reviewed literature and their suitability for systematic and reproducible searching. Nevertheless, relevant Ghanaian studies indexed exclusively in other databases, local journals, or institutional repositories may not have been captured. This may have introduced some database and publication bias. Therefore, geographic and thematic gaps identified in this scoping review should be interpreted as gaps within the evidence retrieved through the predefined search strategy rather than definitive absence of research in those areas. A further limitation is that some included publications may have originated from the same institutions, parent cohorts, or overlapping participant populations. Although these studies addressed different research questions and were therefore retained, such overlap may increase the apparent representation of particular populations, institutions, or thematic areas within the evidence base. The number of included publications should therefore not be interpreted as representing an equivalent number of entirely independent participant cohorts.

Additionally, the synthesis reflects the current landscape of prostate cancer research in Ghana regardless of methodological quality. Finally, because this review relied on published evidence, publication bias cannot be excluded, as studies reporting statistically significant or favourable findings are generally more likely to be published than studies with null or negative results. Despite these limitations, this review provides the first comprehensive synthesis of prostate cancer research conducted in Ghana and offers an important evidence base for identifying national research strengths, deficiencies, and priorities.

### 4.3. Future Research Directions

The findings of this scoping review highlight several priorities for advancing prostate cancer research in Ghana. First, there is an urgent need for population-based epidemiological studies supported by strengthened cancer surveillance systems and expanded population-based cancer registries to generate nationally representative estimates of incidence, mortality, survival, and disease burden. Such data are essential for informed policy development, effective resource allocation, and evaluation of national cancer control strategies. Future research should extend beyond the current concentration of studies in the Greater Accra and Ashanti Regions by undertaking multicentre investigations across underrepresented regions, particularly the Northern, North East, Savannah, Upper East, Upper West, Oti, Bono East, Ahafo, and Western North Regions. Broader geographical representation would improve understanding of regional disparities in disease burden, healthcare access, and treatment outcomes. Also, while awareness and knowledge have been widely investigated, comparatively few studies have evaluated interventions designed to improve screening uptake, facilitate early diagnosis, or reduce delays in treatment. Future implementation research, pragmatic clinical trials, and community-based intervention studies should therefore assess the effectiveness, feasibility, acceptability, and cost-effectiveness of culturally appropriate screening programmes and health education strategies within the Ghanaian context. Greater attention should also be given to rural populations, which remain poorly represented in the published evidence. Health-services research is needed to examine pathways to diagnosis and treatment, including affordability, referral processes, availability of diagnostic and specialist services, treatment infrastructure, and geographic barriers to care. Prospective studies should further evaluate approaches to early detection and risk assessment, while considering feasibility, potential benefits and harms, cost-effectiveness, and health system capacity.

The review also identifies a need for greater investment in translational and precision oncology research. Although several studies have explored genetic susceptibility and molecular biomarkers among Ghanaian men, further validation of emerging biomarkers, the integration of genomic profiling into clinical practice, and the investigation of ancestry-specific disease mechanisms are warranted to support personalized approaches to prostate cancer prevention and treatment. In addition, potential lifestyle and metabolic correlates of PCa remain underexplored in Ghana. Prospective studies are needed to clarify whether diet, adiposity, physical activity, and metabolic health are associated with PCa incidence, progression, or outcomes in Ghanaian populations. These exposures should not currently be framed as established targets for PCa prevention. Future research should also place greater emphasis on survivorship and quality of life. Although recent qualitative studies have highlighted the psychosocial burden experienced by patients and caregivers, longitudinal investigations are needed to evaluate long-term physical, psychological, sexual, nutritional, and economic outcomes following diagnosis and treatment. Such evidence would support the development of comprehensive survivorship care programmes.

## 5. Conclusions

This scoping review successfully addressed its primary objective of systematically mapping the direction and priorities of prostate cancer research conducted in Ghana. By synthesizing evidence from 47 eligible studies, the review provides the first comprehensive overview of the scope, characteristics, and thematic distribution of prostate cancer research in the country. The findings of this scoping review provide an evidence base for identifying future prostate cancer research priorities in Ghana. Prostate cancer research in Ghana has expanded considerably over the past decade, encompassing diverse areas that include awareness and screening, epidemiology, diagnosis, genetics and molecular biomarkers, treatment outcomes, psychosocial experiences, caregiver burden, health economics, and methodological developments. Despite this growing body of evidence, the research landscape remains uneven. The available studies are concentrated within a limited number of urban tertiary healthcare institutions, particularly in the Greater Accra and Ashanti Regions, and are dominated by observational study designs. Consequently, significant knowledge gaps persist regarding the burden and management of prostate cancer in many parts of the country. Evidence remains particularly limited for population-based epidemiology, implementation and intervention research, survivorship, nutritional and lifestyle determinants, health systems research, and studies evaluating equitable access to screening, diagnosis, and treatment services across diverse geographical settings.

The findings of this review establish a clear evidence base for setting future research priorities in Ghana. Addressing the identified gaps will require strengthened national cancer surveillance systems, multicentre and multidisciplinary collaborations, greater inclusion of underrepresented regions, and increased investment in longitudinal, implementation, and translational research. Such efforts will improve the quality and representativeness of the available evidence while facilitating the translation of research findings into effective clinical practice and public health policy. Overall, this scoping review provides an important reference for researchers, clinicians, policymakers, and funding agencies by identifying both the strengths of the existing evidence base and the areas that require further investigation. Strengthening prostate cancer research across the identified priority areas will be essential for improving early detection, optimizing patient management, reducing geographical disparities in care, and ultimately decreasing the burden of prostate cancer among Ghanaian men.

## Figures and Tables

**Figure 1:**
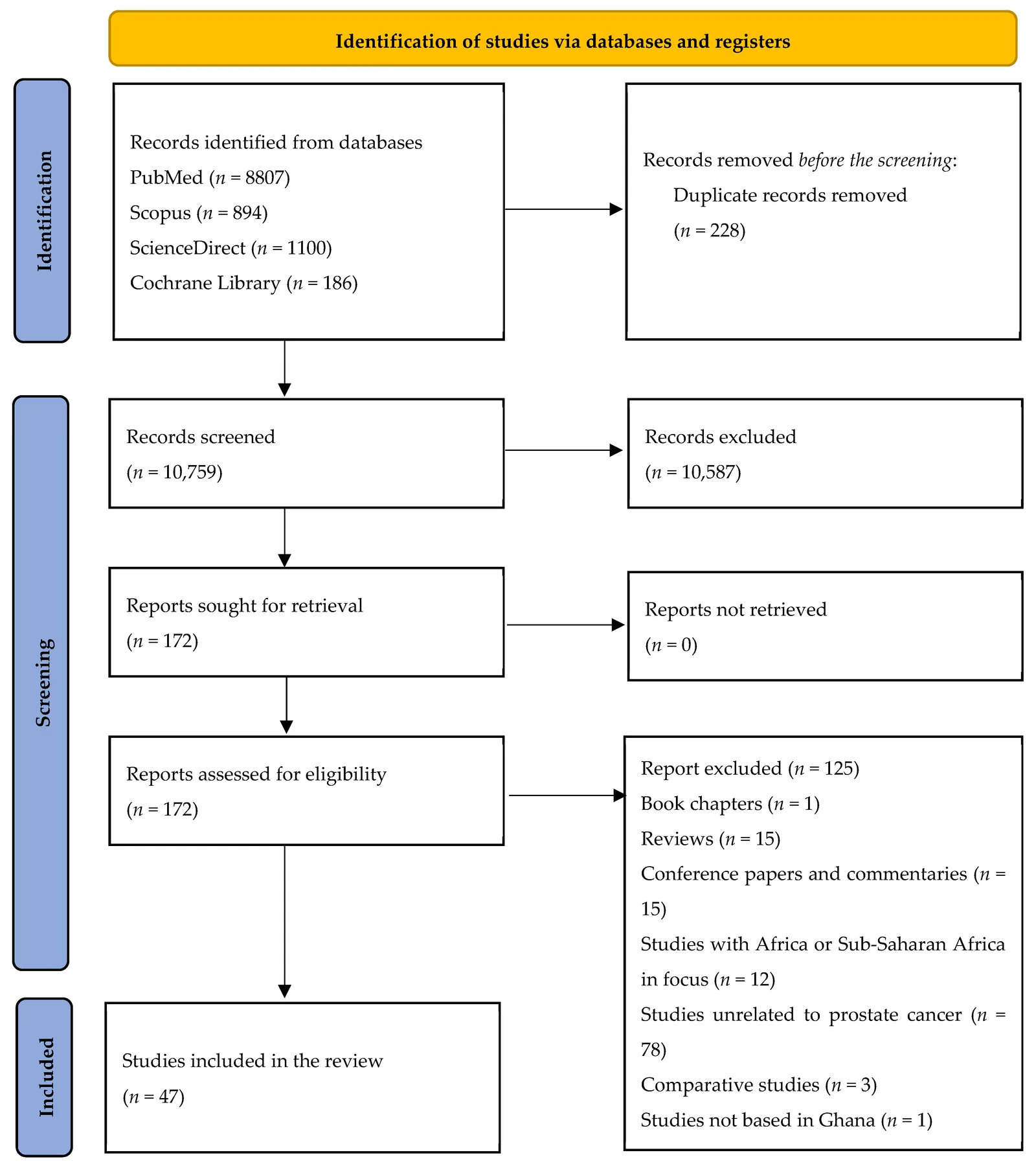
Flowchart summarizing studies evaluated and selected for the scoping review.

**Table 1:** Knowledge, awareness and perceptions of people about prostate cancer (PCa).

Authors Names, Year	Region of Study	Study Design	Sample Size (*n*)	Participants Characteristics	Objective of Study	Outcome of Assessment	Findings
Osei Agyemang et al. [19]	Ashanti Region, Ejisu	Quantitative study	423	Men aged 30 years and above	To assess PCa awareness, knowledge, and screening practices in men aged at least 30 years	A structured questionnaire to assess participants’ PCa awareness, knowledge, and screening status	85.8% of the participants were aware of PCa, while 60.4% knew that PCa affects men only. 50.1% knew the signs and symptoms of PCa; however, 32% did not know about the risk factors of PCa. 67.4% of the participants agreed that PCa can be prevented, but 54.1% did not know any PCa treatment method. 30% and 21.6% of the participants pointed to surgical operations and herbal medicine as treatment, respectively. Although 63.6% have heard of PCa screening, close to 90% have never screened.
Asiamah-Asare & Ackumey [20]	Bono Region, Sunyani	Cross-sectional study	160	Public elementary and high school teachers aged 45 years and above	To assess the awareness and knowledge of PCa among male teachers in the Sunyani municipality of Ghana	A structured questionnaire to assess participants’ knowledge of PCa	88.1% and 78.8% could identify at least one sign and symptom, and risk factors, respectively. 70.6% indicated PCa management via surgery, but 37.5% knew about a screening test for PCa. Overall, 57.5% had adequate knowledge about PCa.
Yeboah-Asiamah et al. [21]	Bono Region, Sunyani municipality	Cross-sectional study	160	Primary, Junior High, and Senior High School teachers, aged 45 and above	To examine perceptions and knowledge about PCa and attitudes towards screening in male teachers in the Sunyani Municipality	Structured questionnaire on characteristics of respondents, perceptions of PCa, and attitudes towards screening	95% agreed that screening for PCa is beneficial; however, 90% had never been screened. 72% and 49.3% disagreed with the assertion that PCa screening is embarrassing and painful, respectively. While 58.8% considered PCa not a sexually transmitted disease, 40.6% attributed it to mobile phone radiation. 93.8% considered PCa as fatal, and 46.2% indicated that one is likely to get PCa at age 50.

**Table 2:** Tools for assessing the knowledge, awareness and perceptions of people about prostate cancer (PCa).

Authors Names, Year	Region of Study	Study Design	Sample Size (*n*)	Participants Characteristics	Objective of Study	Outcome of Assessment	Findings
Wiafe et al. [9]	Ashanti Region, Kumasi, Kejetia Market	Cross-sectional quantitative study	400	Women aged 18 years and above	To conduct an external pilot survey to investigate the practicability of the Akan tool in studying PCa awareness in women	A specialized questionnaire to assess the awareness of PCa in women	None of the knowledge items on the signs and symptoms, and risk factors of the disease had correct responses from more than 25% and 20% of the participants, respectively, while knowledge items on the causes of PCa received varied responses.
Wiafe et al. [22]	Ashanti Region, Agogo (Agogo new community market)	Cross-sectional study	500	Permanent market women aged 18 years or more	To perform a psychometric study on a 41-item tool (PCKAB-Gh) to improve robustness	Akan (Asanti Twi) tool originally developed from several survey questionnaires using forward and backward translation techniques	The indices show that the 5-factor (theme) structure proposed matches the actual data patterns very well, while most questions are strong indicators of their factors (themes). Overall, the tool is statistically valid and robust.

**Table 3:** Sources of prostate cancer (PCa) awareness.

Authors Names, Year	Region of Study	Sample Size (*n*)	Participant Characteristics	Source
Asiamah-Asare & Ackumey [20]	Bono Region, Sunyani	160	Public elementary and high school teachers aged 45 years and above	68% from television whereas 57% from radio.
Osei Agyemang et al. [19]	Ashanti Region, Ejisu	423	Men aged 30 years and above	52.1% from radio, 22.6% from television, 7.7% a friend, 7.4% read about it, 4.6% from a doctor, 4% a relative, 1.6% a nurse.
Daniels et al. [23]	Greater Accra Region, Accra	63	Patients treated with or receiving definitive radiotherapy treatment aged between 50 and 81	Health professionals (89%), friends (62%), television (61%), family members (39%) and internet (48%).

**Table 4:** The cost of prostate cancer (PCa) treatment in Ghana.

Authors Names, Year	Region of Study	Study Design	Sample Size (*n*)	Facilities Characteristics	Objective of Study	Outcome of Assessment	Findings
Wiafe et al. [24]	Ashanti Region	Prevalence-based cost approach	16	Two (2) hospitals, four (4) private laboratories, and ten (10) private community pharmacies	To investigate the cost of PCa diagnosis and management, the availability and prices of PCa medications, and the affordability of PCa care in Ghana	Validated data collection instruments administered to representatives of the selected hospitals and laboratories	Metastatic PCa has a direct cost of diagnosis estimated at GHC 6876.00, whereas non-metastatic PCa costs GHC 1686.00. The estimated cost for PCa management with radical prostatectomy is GHC 2150.00, whereas EBR costs GHC 2150.00. The mean availability of PCa drugs ranged from 41.54–61.54%. The MDPR of PCa medications ranged from 0.72 to 15.38. A 6-month androgen deprivation therapy employing goserelin was GHC 3000.00.

Keywords: EBR—External Beam Radiotherapy; MDPR—Median Drug Price Ratio.

**Table 5:** Barriers and facilitators towards the acceptance of prostate cancer (PCa) screening.

Authors Names, Year	Region of Study	Study Design	Sample Size (*n*)	Participants Characteristics	Objective of Study	Outcome of Assessment	Findings
Yeboah-Asiamah et al. [21]	Bono Region, Sunyani municipality	Cross-sectional study	160	Primary, Junior High and Senior High School teachers, aged 45 and above	To examine perceptions and knowledge about PCa and attitudes towards screening in male teachers in the Sunyani Municipality	Structured questionnaire on characteristics of respondents, perceptions of PCa, and attitudes towards screening	Routine medical examination, PCa awareness activities such as talks on TV and radio, flyers on notice boards, and SMS circulations can promote PCa screening.
Boafo et al. [25]	Greater Accra, Accra Metropolitan Area	Cross-sectional study	356	Convenience sampling of employed, unemployed and retired persons aged 40 years and above	To explore the prevalence of PCa screening among Ghanaian men and interrogate why some individuals screen for the disease and others do not	Health Belief Model (HBM) questionnaire	79.2% of respondents reported being willing to screen for PCa if a healthcare provider recommended it, whereas those who do not know any family member with PCa were less likely to discuss and screen for it. Men in the sample generally did not consider themselves susceptible to PCa.
Maison et al. [26]	Central Region, Cape Coast	Prospective cross-sectional study	282	Men using traditional medicine for urinary symptoms and men living with PCa who had previously sought treatment from traditional healers	To examine the barriers that delay the pursuit of orthodox medical care for PCa in Ghana’s Central Region	A questionnaire that assessed participants’ knowledge of PCa, their attitudes toward PCa screening, and the barriers to seeking orthodox medical care	75% knew of PCa, but only 35% understood it affects males. 45% acknowledged age as a risk factor, while 15.6% attributed PCa incidence to a spiritual cause. 4.3% acknowledged the role of PCa screening in early diagnosis. 61.7% perceived orthodox medical care as more expensive than traditional care. 15.6% acknowledged that religious or cultural beliefs influence healthcare choices.

**Table 6:** Prostate cancer (PCa) management and treatment outcomes.

Authors Names, Year	Region of Study	Study Design	Participants Characteristics	Objective of Study	Findings
Mensah et al. [27]	Greater Accra, Accra	Prospective cohort study	90 patients who underwent brachytherapy alone or brachytherapy with EBR, with a median follow-up of 58 months	To present the experience of a brachytherapy team in Ghana with a focus on technology transfer and outcomes	12 patients experienced biochemical failure, including one patient who died due to metastatic disease. Freedom from biochemical failure rates for low, intermediate, and high-risk cases were 95.4%, 90.9%, and 70.8%, respectively. 76.6% experienced self-limiting grade 1 urinary symptoms, four needed catheterisations, and one had grade 3 urinary symptoms. Out of 84%, 62% reported good erectile function, while 8% had no erection.
Asamoah et al. [28]	Greater Accra, Accra	Retrospective cohort Study	1074 patients with localized and metastasized PCa with a median follow-up of 43 months	To present a review of the management of PCa at the cancer centre of the largest tertiary referral facility in Ghana, with emphasis on curative treatment	High-risk disease presentation was 64.4%. 57.6% of patients with localized disease received curative radiotherapy. The 5-year overall survival for the curative cohort was 96%. The 5-year bDFS rates for low-, intermediate-, and high-risk groups were 95%, 70%, and 48%, respectively.
Daniels et al. [29]	Greater Accra, Accra	Cross-sectional study	122 patients with localized PCa with a median follow-up of 33.1 months	To retrospectively assess the clinicopathological characteristics and biochemical outcomes of patients treated for localized PCa with external beam radiotherapy (EBRT) between 2015 and 2020 at a major cancer treatment centre in Accra, Ghana	86.9% of the patients were free of biochemical progression, whereas 13.1% experienced BCF after receiving EBRT. Erectile dysfunction increased from 1.6% to 18.9% after treatment. Decrease in the number of patients with lower urinary tract symptoms from 73.8% to 45 36.9%.
Yamoah et al. [30]	Greater Accra, Accra	Retrospective cohort study	3798 patients with a biopsy-proven diagnosis of prostate adenocarcinoma with a median follow-up of 38.9 months	To evaluate disease presentation and early clinical outcomes among Ghanaian men with PCa treated with external beam radiotherapy	69.6% had initial PSA greater than 20 ng/mL. 33.8% were diagnosed with metastatic disease at the time of referral. 3- and 5-year actuarial FFbF after EBRT treatment was 73.8% and 65.1%, respectively.
Kyei et al. [31]	Greater Accra, Accra	Prospective study	20 patients with clinically localized PCa with a median follow-up of 19.5 months	To assess early outcomes of a single open radical retropubic prostatectomy procedure after an SIU fellowship	60% of participants were T1c and 40% T2a, with a mean Gleason score of 6.5. The mean estimated blood loss was 1140.0 mL with a transfusion rate of 70%. For the pathologic stage, pT2 cancers formed 60%, pT3 25%, and pT4 5%, with a mean Gleason score of 6.8. The perioperative complication rate was 15%, and the postoperative potency recovery rate was 78.6%, with all the patients being continent of urine. The total PSA of 95% of the patients remained less than 0.4 ng/mL.

Keywords: PD5I—Phosphodiesterase type 5 inhibitors; EBRT—External Beam Radiation Therapy; bDFS—biochemical disease-free survival; BCF—biochemical failure; PSA—prostate-specific antigen; FFbF—freedom from biochemical failure; SIU—The Société Internationale d’Urologie.

**Table 7:** Dosimetric comparison of radiotherapy techniques for prostate cancer (PCa).

Authors Names, Year	Region of Study	Study Design	Sampling Method	Participants Characteristics	Objective of Study	Findings
Kyei et al. [32]	Greater Accra, Accra	Cross-sectional study	Simple random sampling	80 patients with localized disease	To compare the dosimetric properties of 3DCRT and IMRT treatment plans for the external beam irradiation of patients with PCa to ascertain the superiority of IMRT in terms of dose homogeneity, conformity and dose limitation to organs at risk (OAR) in a resource-limited setting	IMRT had better organ sparing compared to 3DCRT. The mean doses for the OARs ranged from 4.3–74.6 Gy for IMRT and 3.1–75.9 Gy for the 3DCRT technique. IMRT had a significantly better mean conformity and homogeneity index compared to 3DCRT. The mean homogeneity indices for the IMRT and 3DCRT treatment techniques ranged from 0.01–0.011 and 0.04–0.016, respectively.

Keywords: 3DCRT—three-dimensional conformal radiotherapy; IMRT—intensity-modulated radiotherapy.

**Table 8:** Psychosocial impact on people living with prostate cancer (PCa).

Authors Names, Year	Region of Study	Study Design	Sample Size (*n*)	Participants Characteristics	Objective of Study	Outcome of Assessment	Findings
Salifu et al. [33]	-	Qualitative study	46	Men with advanced PCa and their caregivers	To explore how advanced PCa impacts men’s masculine identity from the perspective of patients and their caregivers	Consolidated Criteria for Reporting Qualitative Studies (COREQ) was employed	Over half of the men expressed impact on their masculinity due to changes in physical appearance, decreased heterosexual performance, and changes in sociocultural and economic role of participants. Changes in masculinity were associated with emotions of fear and weakness. The caregivers attested to these impacts of PCa on the patients.
Daniels et al. [23]	Greater Accra Region, Accra	Descriptive longitudinal study	63	Patients treated with or receiving definitive radiotherapy treatment aged between 50 and 81	To explore the perceptions of PCa patients on the effects of the disease and radiation therapy on male sexual function	A structured questionnaire on the changes in sexual function from the time of diagnosis through the commencement of treatment till the end	The participants perceived the effects of PCa on sexual function as decrease in sexual desire (54%), premature ejaculation (49%), and decreased sexual activity (48%). The perceived effects of radiotherapy included erectile dysfunction (49%), decreased sexual desire (38%), and premature ejaculation (37%).
Ntiamoah & Senoo-Dogbey [12]	Volta Region, Sogakope and Hohoe	Cross-sectional analytical study	205	Randomly sampled patients diagnosed with PCa	To assess the health-related quality of life and its predictors among patients diagnosed with PCa in two municipalities in the Volta Region of Ghana and to compare the scores between patients receiving treatment and those not receiving treatment	The Functional Assessment of Cancer Therapy-Prostate (FACT-P) Scales, version 4	The participants had an overall lower health-related quality of life with a cancer therapy-prostate total score of 72. Emotional wellbeing was significantly lower among treated patients compared to untreated patients. Treated patients showed marginally better social and functional wellbeing. Old age, erectile dysfunction, bowel problems, and anxiety are predictors of poorer scores.
Annan et al. [34]	Greater Accra, Accra	Qualitative exploratory descriptive study	10	Unmarried men with PCa who receiving treatment at the OPD	To explore the psychological wellbeing and coping strategies of unmarried men with PCa in the Accra Metropolis of Ghana	A semi-structured interview guide	Participants exhibited psychological symptoms of anxiety, depression, distress and suicidal ideation. Depression was associated with frequent urinary retention, severe pain and indwelling catheter, erectile dysfunction and cost of treatment. The participants coped with the condition via lifestyle modification, social contact, and acceptance.

**Table 9:** Psychosocial impact on the caregivers of persons living with prostate cancer (PCa).

Authors Names, Year	Region of Study	Study Design	Sample Size (*n*)	Participants Characteristics	Objective of Study	Outcome of Assessment	Findings
Ninnoni & Owoo [35]	Central Region, Cape Coast	Descriptive study	12	Family caregivers of persons living with PCa	To explore the psychosocial experience of caregiving on the family caregiver of patients with PCa in the Cape Coast metropolis of Ghana	In-depth face-to-face semi-structured interviews	Participants expressed anxiety mainly due to the financial constraints, changes in patients’ physical appearance and news of cancer and its treatment. The participants experienced turmoil, poor quality of sexual life, role adjustment and reduced leisure activities.
Appiah et al. [36]	Greater Accra Region, Accra	Qualitative exploratory study	35	Spouses of men with PCa	To explore the experiences of spouses of men with PCa in Ghana	In-depth face-to-face interviews with a semi-structured guide	Most participants experienced physical strain such as sleeplessness, exhaustion, decreased appetite, leg pain and discomfort, decreased leisure time and financial constraints. Many expressed fears of losing their partners and anxiety about medical procedures, such as catheterisation and the potential infertility and diminished sexual performance of their partners. Participants exhibited variability in coping with PCa.
Owoo et al. [37]	Central Region, Cape Coast	Descriptive study	12	Family caregivers	To explore the challenges encountered by family caregivers of people with PCa	Consolidated Criteria for Reporting Qualitative Studies (COREQ)	Most of the participants reported inadequate knowledge and education about prostate cancer and expressed a need for more information and guidance to support them in caring for patients. Others expressed confusion due to a lack of education about the condition and treatment. Participants reported panic and fear from misconceptions about PCa. All participants reported financial difficulties due to treatment cost and transportation fares. Inadequate hospital facilities and assistance from the health workers were a major challenge for the participants.
Salifu et al. [38]	-	Qualitative study	46	Men living with advanced PCa (23) and caregivers (23)	To explore palliative and end-of-life care experiences of family caregivers and patients living at home in a resource-poor context in Ghana	Interviews with a semi-structured guide	Caregivers expressed a limited access to healthcare support and inadequate knowledge about disease progression. As a result, they often felt overburdened by having to undertake caregiving responsibilities that would ordinarily require professional healthcare support.
Owoo et al. [39]	Central Region, Cape Coast	Descriptive study	12	Family caregivers	To explore how caring affects the physical health of family caregivers of PCa patients	semi-structured face-to-face, in-depth interviews	Most participants experience sleeplessness due to patients’ needs and their concerns for the patient. The participants experienced physical pain as a result of their newly assumed role and expressed an altered dietary pattern leading to weight loss.

**Table 10:** Risk factors and prostate cancer (PCa) diagnosis.

Authors Names, Year	Region of Study, City	Study Design	Sample Size (*n*)	Participants Characteristics	Objective of Study	Findings
Yarney et al. [40]	Greater Accra, Accra	Retrospective study	170	Patients with biopsy-proven diagnosis of carcinoma of the prostate from 2003–2007	To study the clinicopathologic features of PCa in Ghana, in order to determine the factors responsible for them and to find out if there is any relationship between them	73.7% presented with an initial PSA greater than 20 ng/mL, 12.2% had PSA between 10 and 20 ng/mL, whilst 14.1% had PSA less than 10 ng/mL. 56% had a Gleason score ≥ 7. Asymptomatic patients constituted 24.0%; the rest had bone pain (22.6%), urinary (50.4%), and neurologic symptoms (3.0%). A high-fat diet and low intake of fruits and vegetables are probable risk factors in Ghana.
Bosson-Amedenu et al. [41]	Greater Accra, Accra	Retrospective study	300	Men diagnosed with PCa from 2015 to 2020	To evaluate 300 men aged 25–84 years diagnosed with PCa drawn from a major tertiary care centre in the region (2015–2020), to determine how competing health risks influence cancer-specific mortality	Most patients were diagnosed with stage 2 cancer, with a mean cancer size of 5.84 cm and mean lymph node involvement of 9.15 nodes. The mean PSA level was 2.62 ng/mL, and the mean Gleason score was 7.47. 50% of patients received chemotherapy; a similar proportion underwent hormonal therapy, whereas 54% received radiation therapy. Traditional survival estimators are limited in their robustness compared to competing risk models such as the Fine–Gray model in settings with high competing mortality.
Yeboah et al. [42]	Greater Accra	Prospective study	669	Men with PCa from 2004 to 2013	To study the clinical incidence of PCa, risk factors, TNM stage, their management and outcomes	62% had localized disease cases, 25% had locally advanced cases, and 13% had metastasized disease. 67% were at high risk, with Gleason score ≥ 7, and 32.3% were at low and intermediate risk, with Gleason score ≤ 6. 13.8%, 10.5% and 23% of cases were managed by radical prostatectomy, brachytherapy and EBRT, with mortality rates of 0.3%, 0.1% and 0.7%, respectively. All locally advanced cases (25%) received neoadjuvant hormonal therapy, then 0.4% received brachytherapy, and 9.5% received EBRT, with mortality rates of 0.15% and 0.59%, respectively. Locally advanced cases (25%) and metastatic cases (13%) were managed by hormonal therapy alone at mortality rates of 5% and 6%, respectively.
Obeng et al. [43]	Greater Accra, Accra	Cross-sectional study	258	Patients with histologically confirmed adenocarcinoma of the prostate, who were treatment-naïve and undergoing a staging bone scan investigation	To assess the predictive value of prostate-specific antigen (PSA) for bone metastasis and the role of serum ALP in enhancing prediction	Among participants with more than 30% of their core-biopsy volume involved with adenocarcinoma, 54.0% had bone metastasis. 57% of those with perineural invasion had bone metastasis; however, perineural invasion was not a statistically significant predictor. All patients with cribriform pattern histology had bone metastasis. 48.4% of those with bone pain had bone metastasis. ALP, ISUP, and DRE stage significantly improved the bone metastasis detection rate of PSA in PCa.
Tuffour et al. [44]	Greater Accra, Accra	Experimental study	210	Men and women in schistosomiasis endemic community	To ascertain the oncogenic potential of *Schistosoma* spp. soluble egg antigen in human prostate cells using in vitro cellular and biochemical approaches	12.5 μg/mL Soluble egg antigens (SEA) from *S. haematobium* and *S. mansoni* cause prostate cell proliferation. SEA dose-dependently diminished apoptosis significantly in prostate cells.
Hurwitz et al. [45]	Greater Accra, Accra	Case-control study	1031	Men screened for PCa	To examine the relationship between obesity and PCa using a case-control study conducted in Accra, Ghana from 2004 to 2012	Men with BMI ≥ 30 kg/m^2^ had an increased risk of PCa relative to men with BMI < 25 kg/m^2^. Elevated waist circumference and waist-hip ratio were also associated with PCa. Associations were not modified by smoking status and were evident for low- and high-grade disease.
Mensah et al. [46]	Greater Accra, Accra	Retrospective study	7338	Men who underwent TRUS-guided prostate biopsies from July 2005 to December 2022	To determine the role of serum PSA levels and DRE in predicting the histological outcomes of prostate biopsies by analysing a database of over 7000 patients who underwent TRUS-guided prostate biopsies	Out of the 2292 cases diagnosed with cancer, 508 (22.8%) were classified as low-grade and 1784 (77.2%) were classified as high-grade. The prevalence of cancer was lowest in the PSA < 4 ng/mL group at 21.6% and highest in the PSA 100+ ng/mL group at 92.0%. Higher DRE stages correspond to an increased probability of having cancer, with a progressive increase with increasing PSA level. As PSA level increased, the proportion of low-grade cases decreased, and the prevalence of high-grade cancers increased.
Mensah et al. [47]	Greater Accra, Accra	Prospective and retrospective study	234	Men who died of PCa from 2013 to 2022	To describe the characteristics and natural history and treatment received by patients dying of PCa in Ghana	ADT was the predominant treatment, with bilateral orchidectomy performed for 83.9% of patients. Local curative treatment in the form of radical prostatectomy, external beam radiotherapy and brachytherapy was provided to 6% of patients, while palliative radiation therapy was administered to 13%. 51.3% died within 24 months of diagnosis, 23.0% died between 2 and 5 years after diagnosis, while a quarter of the cohort survived for more than 5 years after diagnosis.
Adler et al. [48]	-	Case-control study	1713	Participants aged 53 years or older, recruited through either a population-based probability sample or clinical recruitment.	To evaluate the association between usual adult occupation and PCa risk in Ghanaian men, a population with historically low rates of PCa screening	No association between PCa and regular employment such as motor vehicle operator or farming occupations; however, men in management occupations had increased odds of having PCa. Men in military occupations had higher odds of PCa, both for overall and aggressive PCa, compared with men who were not regularly employed in military-specific occupations.
Obeng et al. [49]	Greater Accra, Accra	One-year cross-sectional review	100	Newly diagnosed patients with PCa with confirmed skeletal metastases, aged 40 years and above	To characterize the anatomical distribution and burden of skeletal metastases in a cohort of newly diagnosed patients with PCa with bone involvement	The axial skeleton accounted for 68.1% of all metastatic deposits involving the spine (32.6%), ribs (25.7%), and pelvis (16.1%), the skull (5.2%), but hand and foot bones had zero metastases. The combination of age (55–75 years), PSA > 100 ng/mL, and ALP ≥ 222 U/L sharply elevates the likelihood of polyostotic disease regardless of already-high clinical risk scores.

Keywords: DRE—digital rectal examination; PSA—prostate-specific antigen; TRUS—transrectal ultrasound; ADT—androgen deprivation therapy; ALP—alkaline phosphatase; ISUP—International Society of Urological Pathology.

**Table 11:** Biomarkers and diagnostic approaches for prostate cancer (PCa).

Authors, Year	Region of Study	Study Design	Sample Size (*n*)	Participant Characteristics	Objective of Study	Diagnostic Tools/Markers Assessed	Findings
Obeng et al. [43]	Greater Accra, Accra	Cross-sectional study	258	Treatment-naïve patients with histologically confirmed prostate adenocarcinoma undergoing staging bone scan investigation	To assess the predictive value of prostate-specific antigen (PSA) for bone metastasis and determine whether serum alkaline phosphatase (ALP) improves prediction	PSA, ALP, DRE stage, ISUP grade, core-biopsy histopathology and staging bone scan	Among patients with >30% core-biopsy involvement by adenocarcinoma, 54.0% had bone metastasis. Bone metastasis was present in 57% of those with perineural invasion and 48.4% of those with bone pain. All patients with cribriform pattern histology had bone metastasis. ALP, ISUP grade and DRE stage significantly improved the bone metastasis detection performance of PSA.
Mensah et al. [46]	Greater Accra, Accra	Retrospective study	7338	Men who underwent TRUS-guided prostate biopsies between July 2005 and December 2022	To determine the roles of serum PSA levels and DRE in predicting the histological outcomes of prostate biopsies	PSA, DRE, TRUS-guided prostate biopsy and histopathological grading	Of 2292 cancer cases, 508 (22.8%) were classified as low-grade and 1784 (77.2%) as high-grade. Cancer prevalence increased from 21.6% among men with PSA < 4 ng/mL to 92.0% among those with PSA ≥ 100 ng/mL. Higher DRE stages were associated with an increased probability of cancer, while increasing PSA levels were accompanied by a greater proportion of high-grade cancers.
Yeboah et al. [50]	Ashanti Region, Kumasi	Cross-sectional prospective study	241	Patients suspected of having a prostate disorder	To develop a prostate disease nomogram for predicting prostate biopsy outcomes by correlating clinical indicators and diagnostic parameters among Ghanaian men	PSA, PSA density (PSAD), DRE, prostate biopsy and multivariable nomogram	PCa was diagnosed in 26.1% of participants. Age, DRE, PSA, PSAD, smoking history and alcohol consumption history were significant independent predictors of PCa. The nomogram provided better prediction of positive prostate biopsy outcomes than the conventional use of PSA, DRE and PSAD alone.
Opoku Mensah et al. [51]	Greater Accra, Accra	Cross-sectional study	237	Patients aged ≥40 years with a suspected prostate disorder	To evaluate the diagnostic and prognostic potential of urinary PCA3 as an alternative biomarker for PCa in the Ghanaian population	Urinary PCA3, PSA and DRE	PCa was diagnosed in 26.6% of participants. PCA3 showed better diagnostic accuracy than PSA or DRE when each was used individually. Combining DRE with PCA3 produced the best diagnostic performance, with sensitivity and specificity of 60.3% and 80.5%, respectively.
Appiah et al. [52]	Bono Region, Wenchi	Case-control study	90	30 men diagnosed with PCa, 30 men with benign prostatic enlargement (BPE), and 30 apparently healthy controls without prostate-related disorders	To explore the relationship between serum PSA, ferritin and hepcidin as new and complementary diagnostic biomarkers for prostate disease	PSA, serum ferritin and serum hepcidin	Serum ferritin and hepcidin levels were significantly higher in patients with PCa than in those with BPE and healthy controls. PSA showed a strong positive correlation with ferritin and a moderate correlation with hepcidin. Serum ferritin showed potential as a diagnostic marker for PCa.
Obeng et al. [53]	Greater Accra	Retrospective cohort study	426	Patients with histologically confirmed PCa	To compare the performance of a multiparametric model with a PSA-only model for predicting metastasis among patients with PCa in Ghana	PSA-only model and multiparametric metastasis-prediction model	The multiparametric model outperformed the PSA-only model across the evaluated performance measures for predicting metastasis in patients with PCa.
Hsing et al. [54]	Greater Accra	Cross-sectional population-based screening study	1037	Apparently healthy men aged 50–74 years	To estimate the burden of PCa among West African men using population-based screening with biopsy confirmation in Ghana	PSA, DRE and prostate biopsy	A total of 352 men had a positive screening result based on PSA or DRE, of whom 307 underwent biopsy. Seventy-three men were confirmed to have PCa, corresponding to a screen-detected PCa prevalence of 7.0%.

Keywords: PSA—prostate-specific antigen; DRE—digital rectal examination; PSAD—prostate-specific antigen density; PCA3—prostate cancer antigen 3; BPE—benign prostatic enlargement.

**Table 12:** Genetics and genomics of prostate cancer (PCa).

Authors Names, Year	Region of Study	Study Design	Sample Size (*n*)	Participants Characteristics	Objective of Study	Findings
Zhou et al. [55]	Greater Accra, Accra	Case-control study	1037	Men aged 50–74 years during 2004–2006 to screen for PCa	To assess the oncoprotein encoded by the *ERG gene* as the established surrogate for *ERG* fusion genes among 262 PCa biopsies from the Ghana Prostate Study	Negative *ERG* staining result was associated with higher Gleason score. The lower prevalence of *TMPRSS2*: *ERG* fusion implies alternative genomic mechanisms might explain the disproportionately high PCa burden in men of African descent.
Chung et al. [56]	-	Experimental study	125	Men of African ancestry, including Ghanaian participants with or without PCa	To perform deep resequencing using NGS in 125 individuals of African ancestry (of which 78 are Ghanaian men) in order to catalogue single nucleotide variants and analyse the genetic architecture of the region	Deep resequencing identified genetic variants across the African-ancestry sample, including 135 novel SNPs reported specifically in the Ghanaian samples and three long non-coding RNAs, RP11-255B23.2, RP11-255B23.3, and RP11-255B23.4, within the targeted 8q24 region (250 kb; chr8:128, 050, 768–128, 300, 801, hg19).
Cook et al. [57]	Greater Accra, Accra	Experimental study	500	Ghanaian men with PCa	To investigate genetic susceptibility to PCa in West Africa	SNPs at 5q31.3 were associated with high Gleason score (7) cancers whilst 6q21 were associated with low Gleason score (<7) cancers. Of the 90 PCa loci reported from other studies of men of European, Asian or African American ancestry, 81 were tested in the Ghana Prostate Study, and 10 were statistically significant.

Keywords: *ERG*—erythroblast transformation-specific–related gene; *TMPRSS2*—transmembrane protease, serine 2, gene; SNPs—single nucleotide polymorphisms.

**Table 13:** Dietary and metabolic factors relevant to prostate cancer (PCa).

Authors Names, Year	Region of Study	Study Design	Sample Size (*n*)	Participants Characteristics	Objective of Study	Findings
Ntim et al. [58]	Ashanti Region, Ayigya	Analytical cross-sectional study	415	Men 40 years or older	To investigate the associations between green tea intake and the serum lipid profile, body mass index (BMI), and serum PSA	Green tea consumption (GTD) was positively associated with improved BMI, a healthier serum lipid profile and lower serum PSA levels, particularly in some older Ghanaian age groups.

**Table 14:** Pharmacological interventions in prostate cancer (PCa) treatment.

Authors Names, Year	Region of Study	Study Design	Cytotoxic Material	Study Model	Objective of Study	Findings
Tettey et al. [59]	Volta Region, Ho	Experimental study	*Croton membranaceus* aqueous root extract	In vitro	To investigate the antioxidative as well as the anti-PCa effects of Vikil^20^	Vikil^20^ demonstrated significant antioxidant effects by suppressing 57.61% and 92.88%, respectively, of DPPH and ABTS radicals. Vikil^20^ suppressed the proliferation of PC-3 cells by reducing the number of viable cells to 49.5%. The migration of PC-3 cells was suppressed prominently within 48 h of incubation with Vikil^20^, and the adhesion of PC-3 cells was significantly suppressed.
Adotey et al. [60]	-	Experimental study	*Ganoderma weberianum-sichuanese*	In vitro	To assess the cytotoxic activity of the mycelial biomass of *Ganoderma weberianum-sichuanese* isolated from the Lower Volta River Basin of Ghana	Fractions of the mycelial biomass significantly inhibit the proliferation and survival of the three cancer cell lines, PC-3, with increasing concentrations.

**Table 15:** Epidemiological measures of prostate cancer (PCa) in Ghanaian studies.

Authors Names, Year	Region of Study	Study Design	Year	Sample Size (*n*)	Participants Characteristics	Objective of Study	Findings
Calys-Tagoe et al. [61]	Greater Accra, Accra	Retrospective cancer registry study	2012	1136	Patients with cancer	To describe the profile of cancers seen at the Korle Bu Teaching Hospital, which is a major referral centre in Ghana for cancers and other health conditions	Overall, there was a cancer prevalence of 274.7 per 100,000 hospital attendees, of which 29.8% were males. 90 of 339 male cancer cases were PCa, representing 26.5% of male cancer cases in the hospital registry.
Amoako et al. [62]	Ashanti Region, Kumasi	Retrospective descriptive study	2015	736	Persons with cancer	To present data on cancers in the city of Kumasi for the year 2015 from the Kumasi Cancer Registry, which is currently the only PBCR in Ghana	A total of 736 cancer cases were recorded, with 37.1 per 100,000 incidences among men. PCa incidence was 10.5 per 100,000 men
Adadey et al. [63]	Volta Region	Retrospective descriptive study	2012–2014	567	Persons with cancer	To review cancer cases recorded in major hospitals in the Volta Region of Ghana, with the aim of determining the incidence of cancers and cancer-related mortalities, and contribute to cancer data in the country	Cancer incidence was approximately 9, 11, and 6 morbidities per 100,000 people in the years 2012, 2013 and 2014, respectively. PCa constituted 16.9% of diagnosed cancer cases in the hospitals reviewed.
Hsing et al. [54]	Greater Accra	Cross-sectional study	2014	1037	Healthy men aged 50 to 74 years old	To estimate the PCa burden in West African men by performing a population-based screening study with biopsy confirmation in Ghana	A total of 352 men had a positive screen by prostate-specific antigen or digital rectal examination, and 307 underwent biopsy. Of these men, 73 were confirmed to have PCa, yielding a 7% screen-detected PCa prevalence among 1037 screened men overall.

## Data Availability

No new data were created or analysed in this study. Data sharing is not applicable to this article.

## Supplementary Materials

The following supporting information can be downloaded at: https://www.mdpi.com/article/10.3390/cancers18203246/s1, Table S1: Preferred Reporting Items for Systematic reviews and Meta-Analyses extension for Scoping Reviews (PRISMA-ScR) Checklist; Table S2: Characteristics of the 47 studies included in the scoping review. References [9,12,19,20,21,22,23,24,25,26,27,28,29,30,31,32,33,34,35,36,37,38,39,40,41,42,43,44,45,46,47,48,49,50,51,52,53,54,55,56,57,58,59,60,61,62,63] are cited in the Supplementary Materials.
