# Supplementary material for: Prostate Cancer Research in Ghana: A Scoping Review of Research Direction and Priorities
Source: Cancers (Basel). 2026 Oct 9;18(20):3246. doi: 10.3390/cancers18203246 (PMC13702399; doi:10.3390/cancers18203246)
Supplement: Supplementary file 1 [file cancers-18-03246-s001.zip › cancers-4511202-supplementary.pdf]

Supplementary Table S1. Preferred Reporting Items for Systematic reviews and Meta-Analyses extension for Scoping Reviews (PRISMA-ScR) Checklist

SECTION	ITEM	PRISMA-ScR CHECKLIST ITEM	REPORTED ON PAGE #
**TITLE**			
Title	1	Identify the report as a scoping review.	Lines 2-3
**ABSTRACT**			
Structured summary	2	Provide a structured summary that includes (as applicable): background, objectives, eligibility criteria, sources of evidence, charting methods, results, and conclusions that relate to the review questions and objectives.	Lines 15-52
**INTRODUCTION**			
Rationale	3	Describe the rationale for the review in the context of what is already known. Explain why the review questions/objectives lend themselves to a scoping review approach.	Lines 113-130
Objectives	4	Provide an explicit statement of the questions and objectives being addressed with reference to their key elements (e.g., population or participants, concepts, and context) or other relevant key elements used to conceptualize the review questions and/or objectives.	Lines 125-130
**METHODS**			
Protocol and registration	5	Indicate whether a review protocol exists; state if and where it can be accessed (e.g., a Web address); and if available, provide registration information, including the registration number.	Lines 132-150
Eligibility criteria	6	Specify characteristics of the sources of evidence used as eligibility criteria (e.g., years considered, language, and publication status), and provide a rationale.	Lines 187-207
Information sources*	7	Describe all information sources in the search (e.g., databases with dates of coverage and contact with authors to identify additional sources), as well as the date the most recent search was executed.	Lines 138-140
Search	8	Present the full electronic search strategy for at least 1 database, including any limits used, such that it could be repeated.	Lines 140-150; Lines 183-186
Selection of sources of evidence†	9	State the process for selecting sources of evidence (i.e., screening and eligibility) included in the scoping review.	Lines 175-214
Data charting process‡	10	Describe the methods of charting data from the included sources of evidence (e.g., calibrated forms or forms that have been tested by the team before their use, and whether data charting was done independently or in duplicate) and any processes for obtaining and confirming data from	Lines 175-180

St. Michael's
Inspired Care.
Inspiring Science.

SECTION	ITEM	PRISMA-ScR CHECKLIST ITEM	REPORTED ON PAGE #
		investigators.	
Data items	11	List and define all variables for which data were sought and any assumptions and simplifications made.	Lines 215-226
Critical appraisal of individual sources of evidence§	12	If done, provide a rationale for conducting a critical appraisal of included sources of evidence; describe the methods used and how this information was used in any data synthesis (if appropriate).	N/A
Synthesis of results	13	Describe the methods of handling and summarizing the data that were charted.	Lines 227-236
**RESULTS**			
Selection of sources of evidence	14	Give numbers of sources of evidence screened, assessed for eligibility, and included in the review, with reasons for exclusions at each stage, ideally using a flow diagram.	Lines 153-174; Lines 238-246
Characteristics of sources of evidence	15	For each source of evidence, present characteristics for which data were charted and provide the citations.	Lines 314-349
Critical appraisal within sources of evidence	16	If done, present data on critical appraisal of included sources of evidence (see item 12).	N/A
Results of individual sources of evidence	17	For each included source of evidence, present the relevant data that were charted that relate to the review questions and objectives.	Lines 314-349
Synthesis of results	18	Summarize and/or present the charting results as they relate to the review questions and objectives.	Lines 314-349
**DISCUSSION**			
Summary of evidence	19	Summarize the main results (including an overview of concepts, themes, and types of evidence available), link to the review questions and objectives, and consider the relevance to key groups.	Lines 350-1387
Limitations	20	Discuss the limitations of the scoping review process.	Lines 1389-1436
Conclusions	21	Provide a general interpretation of the results with respect to the review questions and objectives, as well as potential implications and/or next steps.	Lines 1479-1510
**FUNDING**			
Funding	22	Describe sources of funding for the included sources of evidence, as well as sources of funding for the scoping review. Describe the role of the funders of the scoping review.	Lines 1515-1516

Abbreviations: N/A – Not Applicable

From: Tricco AC, Lillie E, Zarin W, O'Brien KK, Colquhoun H, Levac D, et al. PRISMA Extension for Scoping Reviews (PRISMAScR): Checklist and Explanation. Ann Intern Med. 2018;169:467–473. doi: 10.7326/M18-0850.

Supplementary Table S2. Characteristics of the 47 studies included in the scoping review

Study	Year	Study design	Sample size	Geographic location	Participant characteristics	Principal research domain
Wiafe et al. [9]	2022	Cross-sectional quantitative	400	Kumasi, Ashanti	Women ≥18 years	Knowledge/awareness assessment tools
Ntiamoah & Senoo-Dogbey [12]	2025	Cross-sectional analytical	205	Sogakope and Hohoe, Volta	Patients diagnosed with PCa	Psychosocial impact/quality of life
Osei Agyemang et al. [19]	2024	Quantitative study	423	Ejisu, Ashanti	Men ≥30 years	Knowledge, awareness and screening
Asiamah-Asare & Ackumey [20]	2021	Cross-sectional	160	Sunyani, Bono	Male teachers ≥45 years	Knowledge and awareness
Yeboah-Asiamah et al. [21]	2017	Cross-sectional	160	Sunyani Municipality, Bono	Male school teachers ≥45 years	Perceptions and screening attitudes
Wiafe et al. [22]	2023	Cross-sectional psychometric study	500	Agogo, Ashanti	Adult female market workers	Knowledge/awareness assessment tool
Daniels et al. [23]	2024	Descriptive longitudinal	63	Accra, Greater Accra	PCa patients receiving definitive radiotherapy, 50–81 years	Psychosocial/sexual effects
Wiafe et al. [24]	2022	Prevalence-based cost approach	16 facilities	Ashanti	2 hospitals, 4 laboratories and 10 pharmacies	Cost of PCa care

Study	Year	Study design	Sample size	Geographic location	Participant characteristics	Principal research domain
Boafo et al. [25]	2023	Cross-sectional	356	Accra Metropolitan Area	Men ≥40 years	Screening barriers/facilitators
Maison et al. [26]	2025	Prospective cross-sectional	282	Cape Coast, Central	Men using traditional medicine for urinary symptoms/PCa	Healthcare-seeking barriers
Mensah et al. [27]	2016	Prospective cohort	90	Accra, Greater Accra	PCa patients receiving brachytherapy ± EBRT	Treatment outcomes
Asamoah et al. [28]	2018	Retrospective cohort	1,074	Accra, Greater Accra	Patients with localized or metastatic PCa	Treatment outcomes
Daniels et al. [29]	2023	Cross-sectional/retrospective clinical study	122	Accra, Greater Accra	Localized PCa patients treated with EBRT	Treatment outcomes
Yamoah et al. [30]	2013	Retrospective cohort	3,798	Accra, Greater Accra	Biopsy-confirmed PCa patients treated with EBRT	Treatment outcomes
Kyei et al. [31]	2013	Prospective study	20	Accra, Greater Accra	Patients with clinically localized PCa	Surgical treatment outcomes
Kyei et al. [32]	2024	Cross-sectional dosimetric study	80	Accra, Greater Accra	Patients with localized PCa	Radiotherapy dosimetry

Study	Year	Study design	Sample size	Geographic location	Participant characteristics	Principal research domain
Salifu et al. [33]	2023	Qualitative	46	Ghana; specific region not stated	Men with advanced PCa and caregivers	Psychosocial effects/masculinity
Annan et al. [34]	2024	Qualitative exploratory descriptive	10	Accra, Greater Accra	Unmarried men receiving PCa treatment	Psychological wellbeing
Ninnoni & Owoo [35]	2023	Descriptive phenomenological	12	Cape Coast, Central	Family caregivers of PCa patients	Caregiver psychosocial burden
Appiah et al. [36]	2024	Qualitative exploratory	35	Accra, Greater Accra	Spouses of men with PCa	Spousal/caregiver experiences
Owoo et al. [37]	2022	Descriptive phenomenological	12	Cape Coast, Central	Family caregivers	Caregiver challenges
Salifu et al. [38]	2021	Qualitative	46	Ghana; specific region not stated	23 men with advanced PCa and 23 caregivers	Palliative care/caregiver experiences
Owoo et al. [39]	2022	Descriptive phenomenological	12	Cape Coast, Central	Family caregivers	Physical impact of caregiving
Yarney et al. [40]	2013	Retrospective	170	Accra, Greater Accra	Biopsy-confirmed PCa patients	Clinicopathology/risk factors
Bosson-Amedenu	2025	Retrospective	300	Accra, Greater Accra	Men diagnosed with PCa	Clinicopathology/survival

Study	Year	Study design	Sample size	Geographic location	Participant characteristics	Principal research domain
et al. [41]						
Yeboah et al. [42]	2016	Prospective	669	Greater Accra	Men with PCa	Clinical presentation and management
Obeng et al. [43]	2025	Cross-sectional	258	Accra, Greater Accra	Treatment-naïve, histologically confirmed PCa patients	Biomarkers/metastasis prediction
Tuffour et al. [44]	2018	Experimental	210*	Accra, Greater Accra	Participants from schistosomiasis-endemic setting/prostate-cell experiments	Experimental risk factors
Hurwitz et al. [45]	2020	Case-control	1,031	Accra, Greater Accra	Men screened for PCa	Obesity/adiposity and PCa
Mensah et al. [46]	2025	Retrospective	7,338	Accra, Greater Accra	Men undergoing TRUS-guided prostate biopsy	PSA, DRE and biopsy diagnosis
Mensah et al. [47]	2023	Prospective/retrospective	234	Accra, Greater Accra	Men who died from PCa	Treatment and mortality
Adler et al. [48]	2019	Case-control	1,713	Ghana Prostate Study; region not specified in table	Ghanaian men ≥53 years	Occupational risk factors
Obeng et al. [49]	2025	Cross-sectional review	100	Accra, Greater Accra	Newly diagnosed PCa patients with skeletal	Metastatic disease patterns

Study	Year	Study design	Sample size	Geographic location	Participant characteristics	Principal research domain
					metastases	
Yeboah et al. [50]	2018	Prospective cross-sectional	241	Kumasi, Ashanti	Men with suspected prostate disorders	Diagnostic prediction/nomogram
Opoku Mensah et al. [51]	2022	Cross-sectional	237	Accra, Greater Accra	Men ≥40 years with suspected prostate disorders	PCA3/diagnostic biomarkers
Appiah et al. [52]	2025	Case-control	90	Wenchi, Bono	30 PCa, 30 BPE and 30 healthy controls	Ferritin/hepcidin biomarkers
Obeng et al. [53]	2025	Retrospective cohort	426	Greater Accra	Histologically confirmed PCa patients	Metastasis prediction
Hsing et al. [54]	2014	Population-based cross-sectional screening	1,037	Greater Accra	Apparently healthy men aged 50–74 years	Epidemiology/screen-detected prevalence
Zhou et al. [55]	2017	Case-control/molecular study	1,037 screened; 262 PCa biopsies assessed	Accra, Greater Accra	Ghana Prostate Study participants/PCa biopsy specimens	Genetics/genomics
Chung et al. [56]	2014	Experimental genomic study	125	Mixed African-ancestry sample; Ghanaian subset	125 African-ancestry individuals, including 78 Ghanaian participants	Genetics/genomics

Study	Year	Study design	Sample size	Geographic location	Participant characteristics	Principal research domain
Cook et al. [57]	2014	Genome-wide association study	**474 cases + 458 controls**	Greater Accra/Ghana Prostate Study	Ghanaian PCa cases and population-based controls	Genetics/genomics
Ntim et al. [58]	2025	Analytical cross-sectional	415	Ayigya, Ashanti	Ghanaian men ≥40 years	Dietary and metabolic factors
Tettey et al. [59]	2024	Experimental in vitro	N/A	Ho, Volta	PC-3 prostate cancer cells; *Croton membranaceus* extract	Pharmacological intervention
Adotey et al. [60]	2025	Experimental in vitro	N/A	Lower Volta River Basin, Ghana	PC-3 and other cancer cell lines; *Ganoderma* material	Pharmacological intervention
Calys-Tagoe et al. [61]	2014	Retrospective cancer-registry study	1,136	Accra, Greater Accra	Cancer patients recorded at Korle Bu	Epidemiological measures
Amoako et al. [62]	2019	Retrospective descriptive registry study	736	Kumasi, Ashanti	Persons with cancer in Kumasi Cancer Registry	Epidemiological measures
Adadey et al. [63]	2020	Retrospective descriptive	567	Volta Region	Patients with cancers recorded in major hospitals	Epidemiological measures

Abbreviations: N/A – Not Applicable
